# The effect of epigenetic modifications on the secondary structures and possible binding positions of the N-terminal tail of histone H3 in the nucleosome: a computational study

**DOI:** 10.1007/s00894-017-3308-x

**Published:** 2017-03-28

**Authors:** Louis L. du Preez, Hugh-G Patterton

**Affiliations:** 1 Department of Microbiological, Biochemical and Food Biotechnology, University of the Free State, Bloemfontein, 9301 South Africa; 2 Division of Bioinformatics and Department of Biochemistry, Stellenbosch University, Private Bag X1, Matieland, 7602 South Africa

**Keywords:** Molecular dynamics, Histone H3, Chromatin, Secondary structure, Molecular docking

## Abstract

The roles of histone tails as substrates for reversible chemical modifications and dynamic cognate surfaces for the binding of regulatory proteins are well established. Despite these crucial roles, experimentally derived knowledge of the structure and possible binding sites of histone tails in chromatin is limited. In this study, we utilized molecular dynamics of isolated histone H3 N-terminal peptides to investigate its structure as a function of post-translational modifications that are known to be associated with defined chromatin states. We observed a structural preference for α-helices in isoforms associated with an inactive chromatin state, while isoforms associated with active chromatin states lacked α-helical content. The physicochemical effect of the post-translational modifications was highlighted by the interaction of arginine side-chains with the phosphorylated serine residues in the inactive isoform. We also showed that the isoforms exhibit different tail lengths, and, using molecular docking of the first 15 N-terminal residues of an H3 isoform, identified potential binding sites between the superhelical gyres on the octamer surface, close to the site of DNA entry/exit in the nucleosome. We discuss the possible functional role of the binding of the H3 tail within the nucleosome on both nucleosome and chromatin structure and stability.

**Electronic supplementary material:**

The online version of this article (doi:10.1007/s00894-017-3308-x) contains supplementary material, which is available to authorized users.

## Introduction

The genetic material of eukaryotes is packaged in a repeating oligomeric protein–nucleic acid complex known as chromatin [1]. The fundamental structural unit of chromatin is the nucleosome, which is composed of 168 bp of DNA spooled onto the histone octamer of two copies of each of the core histones H2A, H2B, H3, and H4. A single copy of the linker histone H1 is bound to the outside of the structure, at the point of DNA entry/exit in the nucleosome. The structure of the nucleosome core containing 147 bp of DNA and recombinant histones has been elucidated by X-ray crystallography to a resolution of 1.9 Å [2]. The N- and C-terminal extensions, or histone “tails,” generally dissociate from their binding positions at the ionic strengths employed in crystallization, are not regularly packed in the crystal, and are thus not observed as regions of high electron density [2, 3]. In the absence of observed secondary structures and preferential binding positions, the tails are often described as “unstructured” [3].

These tails are the targets of several post-translational modifications (PTMs) [4], with the tail of histone H3, which is also the longest tail, containing the most targeted residues. PTMs have been shown to be involved in the establishment of an actively transcribed state in decondensed euchromatin [5] and in transcriptional repression in condensed heterochromatin [6]. Lysine acetylation of histone tails is classically associated with euchromatin [7], while lysine methylation of (particularly) H3K9 and H3K27 is largely associated with heterochromatin [8]. More than one modification is often found in multiple tails in a chromatin region, and crosstalk between modifications, such as H3K9me2 and the adjacent S10ph, has been observed [9]. This has led to the proposal that PTMs serve as a code that is deposited by writer proteins and read by reader proteins in order to effect changes in chromatin structure and functional state [10]. Although this hypothesis has been accurate when attempting to describe some processes, such as the recruitment of the heterochromatin-associated protein HP-1 to regions marked by H3K9me3 for transcriptional silencing [11], it has been found lacking in other instances, such as the observation that deacetylation of K16 in histone H4 is required for chromatin compaction in vitro [12]. This underscores our current understanding that the modifications are not merely signals but also participate directly in the dynamics of chromatin structure/functional state transitions. This involvement may take the form of changing the conformation of a modified peptide or protein, as observed elsewhere [13, 14].

However, little attention has been paid to the possible structure of the “disordered” histone N-terminal tails. Biophysical data have hinted at secondary structures being present in the H3 and H4 tails, with CD studies indicating that these tails contribute approximately 30% to the total α-helical content of the nucleosome in an acetylated state [15, 16]. Molecular dynamics (MD) studies have been instrumental in supporting the notion that the H3 tail, in particular, contains secondary structure elements. Earlier simulations on isolated peptides showed hints of α-helical content within the H3 tail. However, these simulations were performed on limited timescales and not on the full-length tail [17, 18]. In the study by Liu and coworkers [18], they established that a shortened H3 tail peptide (residues 1–25) contained an α-helix and also, interestingly, that the dual dimethylation and acetylation of H3K4 and H3K9 had an effect on the stability of the helical content of the peptide. Similarly, Yang and Arya showed in simulations of isolated H4 tail peptides that the α-helical content of the peptide increased following acetylation of K16 [19], hinting that PTMs also played a role in modulating the structure of the histone tails, and presumably that of chromatin as well. More recent simulations in the context of the nucleosome have confirmed earlier suggestions that the H3 tail has a propensity to stabilize α-helical content within the tail [20, 21]. However, those studies focused on nucleosome stability and did not contain modified histone tails.

One key question that remains is how or indeed if the proposed structural content of the H3 tail could affect nucleosome structure and dynamics. Biophysical data have shown that the linker DNA of nucleosomes with hyperacetylated H3 and H4 tails are more mobile [22–25]. Based on the location of the H3 tail at the entry/exit point of the linker DNA and the observed binding of the H4 tail to the acidic patch formed by H2A and H2B in cocrystals [3], it is possible that the H3 tail interacts with the linker DNA and the octamer. This presents the possibility that PTMs may influence interactions of the H3 tail with the nucleosome in the context of chromatin. Additionally, a 15-residue, tail-like viral peptide, Kaposi’s sarcoma herpes virus latency associated nuclear antigen (KSHV LANA), was shown to bind to the acidic patch of the nucleosome [26], presenting the possibility that only a subsection of the H3 tail may be required to bind to the nucleosome.

In the study reported in the present paper, we performed conventional long-timescale, explicit, all-atom molecular dynamics (MD) simulations on experimentally observed PTM isoforms of the full-length histone H3 tail (residues 1–38). We first established the structure of the unmodified tail and whether we could explain the biophysical data associated with a hyperacetylated (K*ac) H3 tail. Next we investigated whether there was a difference in the structure of the tail between a H3 tail modified with an active PTM pattern (K4Me3, K9Ac, K14Ac, K36Me3), i.e., a pattern observed in euchromatin [27], versus an inactive PTM pattern (K9Me2, S10Pho, K27Me2, S28Pho), i.e., a pattern observed in heterochromatin [28]. We found that two α-helices were stabilized in the unmodified H3 isoform, while the two isoforms associated with actively transcribed, decondensed chromatin lacked these helices. We also found that the isoform associated with transcriptionally silenced and condensed chromatin stabilized an α-helix in a different region of the tail.

To place the observed structures in the context of the nucleosome and chromatin, we probed the nucleosome for potential binding sites of the histone H3 N-terminal tail using the 15-residue tips of the simulated tail isoforms. Based on our findings, we propose a potential molecular mechanism for the role of the histone H3 N-terminal tail within the nucleosome which may have implications for higher-order chromatin folding.

## Materials and methods

### Secondary structure prediction

In order to obtain a reference point for the secondary structure composition of the unmodified tail, the secondary structure of the 43-residue H3 N-terminal tail was predicted using the algorithms listed in Table S1 in the “Electronic supplementary material” (ESM).

### Structure preparation

All structure manipulations were performed using the molecular mechanics package YASARA [29]. The highest-resolution X-ray crystal structure available for the nucleosome core particle (NCP) that included the histone tails, 1KX5, was retrieved from the Brookhaven Protein Data Bank (www.rcsb.org), and the first 43 residues of chain A were selected as an independent structure, and represented the unmodified H3 tail.

PTMs were modeled by replacing the native residue in the unmodified tail with a suitably modified residue obtained from NMR and X-ray structures (Table S2 in the ESM). Two peptides were also constructed where all the residues in the unmodified H3 tail were replaced with alanine or glycine residues, which acted as positive and negative control peptides, respectively, for helix formation. All modified peptides used in this study are listed in Table 1.

**Table 1:** Histone H3 tail modifications incorporated into the different isoforms. The experimental sources for the modification patterns are indicated

	Modifications	Reference
Active	K4 + K36me3, K9 + K14ac	[18]
Inactive	K9 + K27me2, S10 + S28ph	[19]
Hyper-Aly	All Kac	[20, 21]
Ala ctrl	All residues are alanine	[22, 23]
Gly ctrl	All residues are glycine	[22, 24]

### Molecular dynamics

Molecular dynamics was performed on all peptides using YASARA [29] and the AMBER03 force field [30] with TIP3P explicit solvation [31]. Residues 39–43 of the H3 tail structure were fixed, and the structure was placed in a rectangular simulation cell with an extension of 16 Å around all atoms. Periodic boundary conditions were used. Simulations were run at 298 K using a weakly coupled Berendsen thermostat [32] and at a pressure of 1 bar utilizing a Solvent Probe pressure control mode. A multiple time step for integration was used, where intramolecular forces were calculated every 2 f. and intermolecular forces every 2.5 fs. The simulation was run at a pH of 7.0 and the cell was neutralized with sodium and chloride counterions to a final concentration of 154 mM [33]. For modified residues, new force field parameters were derived using YASARA Auto SMILES [34–37]. The hydrogen-bond network was optimized to give more stable trajectories. To remove steric clashes and correct the covalent geometry, a structure was energy-minimized with the AMBER03 force field [30] using a 7.86 Å force cutoff. The particle mesh Ewald algorithm [38] was used to treat long-range electrostatic interactions with a force cutoff of 7.86 Å. After removing conformational stress by performing a short steepest-descent minimization, the procedure was continued by implementing simulated annealing (time step 2 fs, atom velocities scaled down by 0.9 every tenth step) until convergence was reached (the energy improved by less than 0.05 kJ/mol per atom during 200 steps). Each system was simulated on the High Performance Computing (HPC) cluster at the University of the Free State for 500 ns on 24-CPU coresingle nodes. Coordinates were saved every 25 ps, yielding 20,000 time points for each trajectory.

### Clustering

All trajectories were converted to xtc format using the YASARA script *md*_*convert*. The GROMACS [39] program *g*_*cluster* was subsequently used to cluster each trajectory, using single-linkage clustering with a 13-Å root mean square deviation (RMSD) cutoff. RMSD values were calculated based on the α-carbons of each structure in each trajectory.

### Secondary structure and hydrogen-bond analysis

The secondary structure and hydrogen bonding present in the histone H3 tail was analyzed using YASARA and in-house Python (http://www.python.org) scripts imported into an in-house-developed simulation results management database, SimDB. Downstream analysis and the generation of figures were performed using this platform. All graphs and figures were generated using the data from the database and graphed using R (http://www.r-project.org).

### Maximum tail length analysis

The maximum sweep-out distance for the H3 tail in each trajectory was calculated using YASARA and in-house Python scripts as follows: for each frame, the α-carbon of P43 was chosen as the reference point, and the distance between it and all other α-carbons in the N-terminal direction of the peptide was calculated. The maximum distance in the list was then selected as the maximum distance for the current frame.

To calculate whether the maximum distance of one tail was significantly different from that of another tail over the total simulation time, an unpaired *t*-test with a Welch correction for unequal variances at a 95% confidence interval was performed and graphed using R.

### Molecular docking

To generate starting structures for molecular docking, the MD trajectories were reclustered as described above, except that the RMSD calculation was based on only the first 15 N-terminal α-carbons.

Rigid molecular docking of the 15-residue histone H3 N-terminal tips was performed using Autodock [40] in YASARA [29]. The nucleosome was divided into overlapping grid cells (Fig. S1 in the ESM). Each grid cell yielded 400 docking poses (ga_run=400, ga_pop_size=400 and ga_eval=10 000), for a total of 3600 docking poses per structure docked.

### Analysis of docking results

Total contacts, hydrophobic contacts, and hydrogen bonds (within 5 Å) between the tip structures and the NCP were identified with YASARA, and Python scripts were used to evaluate and define how the H3 tail contacted the nucleosome.

### Validation of docking results

Each top docked structure used in the analysis above was subjected to a 10-ns MD simulation using the parameters described above to assess the veracity of the docked conformations. The tail tips were completely flexible, while the nucleosome was positionally restrained to prevent the simulation from being prohibitively expensive in terms of computational resources.

### Construction of the tetranucleosome model

To place the tail reach data from the MD simulations and the binding positions from the molecular docking experiments in the context of compact chromatin, we constructed a model based on the tetranucleosome. For the reach data, two spheres were created. The inner sphere had a radius of the reach length of the first quartile of the tail reach data over the entire simulation, while the outer sphere had a radius of the third quartile of the tail reach data. Thus, the volume between the edge of the outer sphere and the edge of the inner sphere represented the area wherein the majority of the tail reach data points were located. The center of this multisphere was placed on P43 of each histone H3. A multisphere was created for each histone H3 in the tetranucleosome, for both the active and inactive tail.

To incorporate the binding positions of the tail tips, the best binding position identified in the molecular docking studies was superimposed onto each nucleosome in the tetranucleosome.

## Results

We have used molecular dynamics (MD) with an all-atom structure and an explicit water model in the presence of physiological concentrations of monovalent ions to investigate the impact of epigenetic modifications on the structure of the histone H3 N-terminal tail. As a starting point, we first applied available structure prediction algorithms to the H3 tail.

### The unmodified histone H3 is predicted to contain two distinct α-helices

Secondary structure prediction tools predicted two α-helices (Fig. S2 in the ESM) in the H3 N-terminal tail. The shorter of the two was situated between K4 and S10, and the longer helix approximately between R17 and R26. The longer helix was predicted by more algorithms, and was flanked by the modifiable K14, K27, and S28 residues. The modified residues thus seemed to be at positions where they could potentially influence the secondary structure of the tail. However, the prediction tools provided one-dimensional information on a limited number of secondary structures. In order to obtain deeper insight into the structural dynamics of the tail, we proceeded with the MD simulations to obtain a three-dimensional simulation of the tail structure.

### The secondary structures in G_43_ and A_43_ are correctly simulated

To confirm that the MD protocol could successfully reflect the secondary structures that are known to be formed in solution, we performed MD simulations of A_44_ and G_44_, which are known to respectively form a stable and a severely disrupted α-helix in solution [41–44]. The evolution of the secondary structures for each peptide are shown in Figs. S3a and S3b in the ESM. The fractional distribution of secondary structures at each residue over a 500-ns simulation run is shown in Figs. S4a and S4b in the ESM. It is clear from these figures that the MD protocol successfully mimics the inherent propensity of peptides for α-helix secondary structures in solution.

### The unmodified N-terminal tail of histone H3 showed a defined secondary structure that is influenced by PTMs

**Fig. 1a–d:**
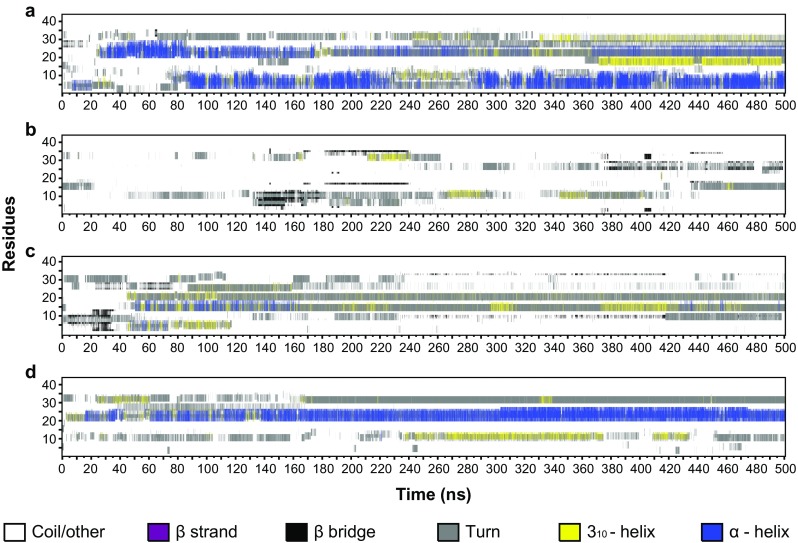
Evolution of the secondary structure content of the unmodified H3 tail (**a**), the hyperacetylated H3 tail (**b**), the active H3 tail (**c**), and the inactive H3 tail (**d**) over the course of a 500-ns all-atom MD simulation. Random coils and other elements are indicated in* white*, β-strands in* purple*, β-bridges in* black*, hydrogen-bonded turns in* gray*, 3_10_ helices in* yellow*, and α-helices in* blue*

**Fig. 2a–d:**
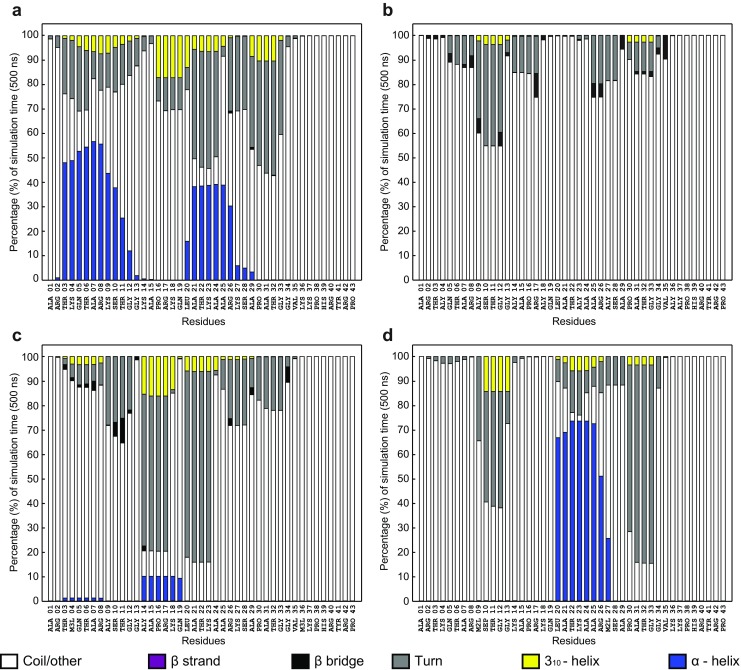
Percentage of simulation time spent in specific secondary structure elements by each residue in the unmodified H3 tail (**a**), the hyperacetylated H3 tail (**b**), the active H3 tail (**c**), and the inactive H3 tail (**d**). Random coils and other elements are indicated in* white*, β-strands in* purple*, β-bridges in* black*, hydrogen bonded turns in* gray*, 3_10_ helices in* yellow*, and α-helices in* blue*

We next investigated the structure of the histone H3 tail peptides listed in Table 1 by using 500-ns explicit all-atom MD simulations. Figure 1 shows the evolution of secondary structures in the H3 peptides during a 500-ns simulation. The unmodified H3 tail stabilized two α-helices: one at the N-terminal tip of the peptide between T3 and G12 and a second in the middle of the tail, between L20 and A29 (Fig. 1a)—positions similar to those predicted by the tools mentioned above. We will refer to these α-helices as the “tip” and the “middle” helix in the text below. Figure 2 shows the fractional distribution of secondary structure elements at each residue for each H3 tail over the 500-ns simulation. It is clear that the tip helix was more stable during the simulation than the middle helix (Fig. 2a).

We next considered the hyperacetylated H3 tail. Although this level of acetylation of the histone tail is not generally observed in situ, it does provide a test of the responsiveness of secondary structures to a defined PTM. Strikingly, the presence of acetyl groups on all the lysine residues completely abolished the formation of the α-helices observed in the unmodified H3 tail. Instead, the periodic appearance of β-bridges involving K9, G12, R17, A25, R26, A29, and V35 (Fig. 1b) were observed. The β-bridges were not particularly stable throughout the entire simulation period (Fig. 2b). Given the significant impact of hyperacetylation on secondary structures in the H3 tail, we proceeded to investigate the effect of biologically relevant PTMs on the tail structures.

The active H3 tail showed the formation of some α-helical content between the acetylated K14 and Q19, although it was not very stable over an extended period in the ns range (Figs. 1c and 2c in the ESM). Some β-bridges were also observed involving residues S10, T11, and G34. Overall, the secondary structure of the active H3 tail was dominated by the formation of 3_10_-helical content and turns. The inactive H3 tail, in contrast, showed the stabilization of an α-helix between L20 and the dimethylated K27, which persisted in the simulation for 60–70% of the total simulation time (Figs. 1d and 2d). This α-helix was accompanied by stable turns between the dimethylated K9 and G13, and between P30 and G33.

### PTM patterns produce unique H3 tail structures

**Fig. 3a–d:**
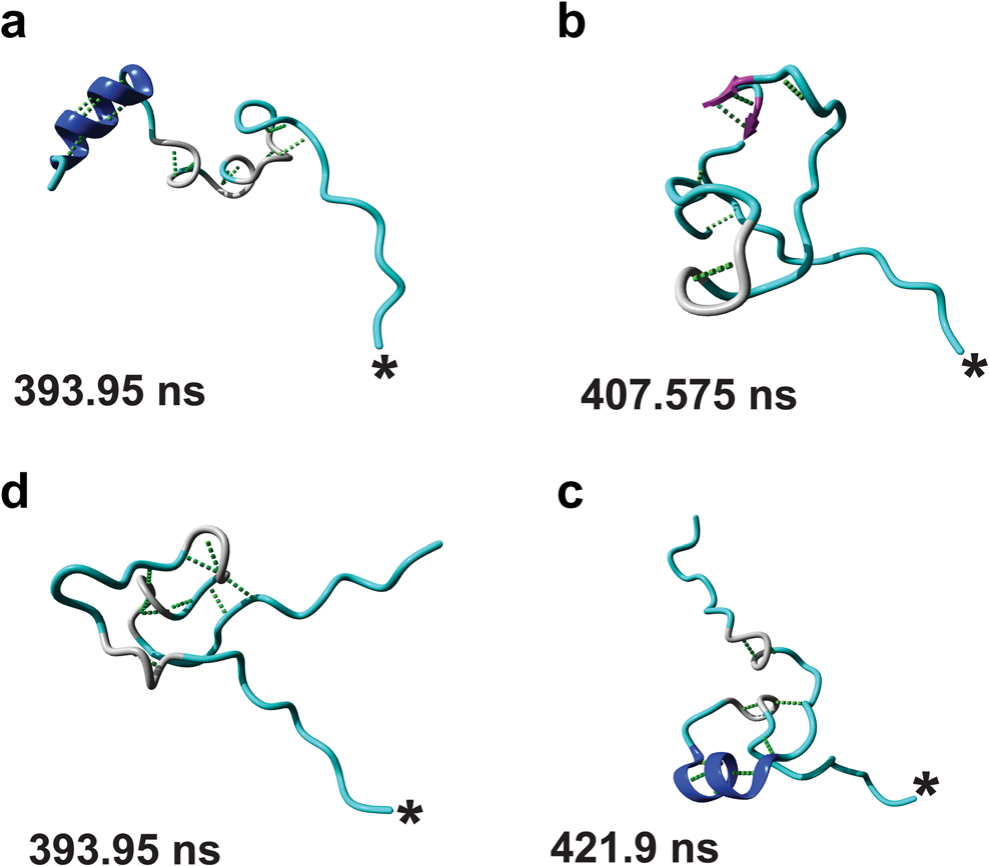
Representative structures of the top most populated clusters found for the unmodified H3 tail (**a**), the hyperacetylated H3 tail (**b**), the active H3 tail (**c**), and the inactive H3 tail (**d**). The* asterisk* indicates the C-terminal of each structure and the time at which the structure occurs is indicated. Random coils and other elements are indicated in* cyan*, β-strands in* purple*, β-bridges in* black*, hydrogen-bonded turns in* gray*, 3_10_ helices in* yellow*, and α-helices in* blue*. Molecular graphics were created with YASARA (www.yasara.org) and POVRay (www.povray.org)

We next investigated the three-dimensional structure of the H3 tails by performing cluster analyses on each of the simulation trajectories. Figure 3 shows the median structure of the most populated structural cluster for each H3 tail during its respective MD simulation. The structures for additional clusters are shown in Fig. S5 in the ESM.

The median structure of the most populated cluster showed an unmodified H3 tail assuming an extended structure, with the tip helix present in all the clusters. The middle helix was only present in the third most populated structure (Fig. 3a and Fig. S5a in the ESM), underscoring its instability relative to the tip helix. Upon hyperacetylation, we observed populations of bulge-like structures stabilized by hydrogen-bonded turns for the majority of the tail (Fig. 3b and Fig. 5b in the ESM), which bears little resemblance to the unmodified structures (Fig. 3a and Fig. S5a in the ESM). Interestingly, we could only obtain one cluster for the active H3 tail that showed extended N-terminal and C-terminal ends with an elaborate bulge-like structure in the middle of the peptide (Fig. 3c). This structure resembled the structures obtained for the hyperacetylated H3 tail, and, upon closer inspection, the bulge was observed to consist of two overlapping loops stabilized by a network of hydrogen bonds. Only two clusters were obtained for the inactive H3 tail, and both showed a more extended structure compared to the preferred active structure, with a stable α-helix and a hydrogen-bonded turn identified near the N-terminal and the C-terminal ends (Fig. 3d and Fig. 5c in the ESM).

### The difference between the structures of the active and inactive H3 tail is mediated by phosphorylated S10, S28, and R17

Next we scrutinized the representative structure of each of the top clusters obtained from the active and inactive H3 tails to understand the basis for their structural divergence. Figure S6 in the ESM shows the occurrence of the hydrogen bonds observed in the clustered structures across the two trajectories.

**Fig. 4a–f:**
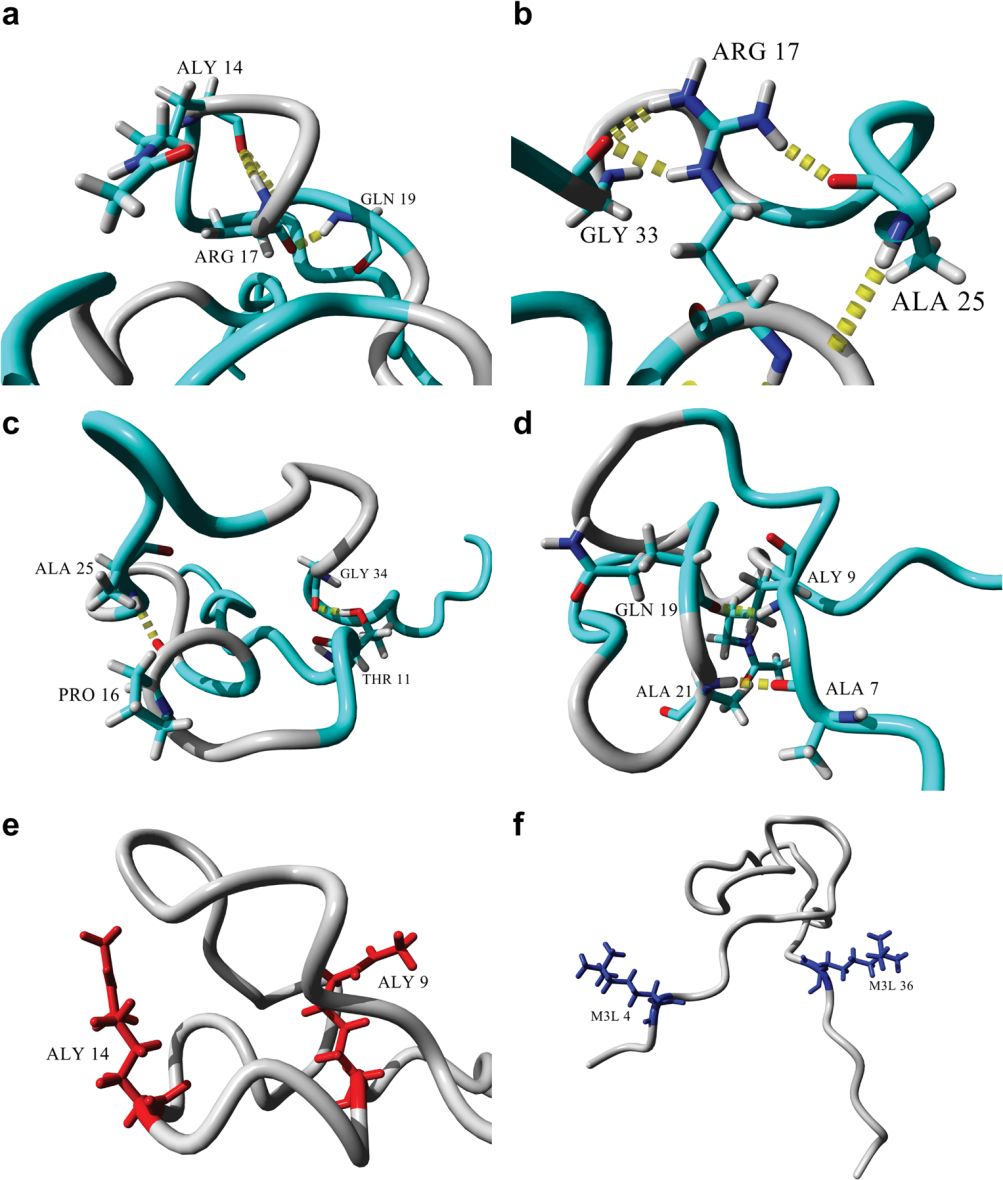
Stabilization of the active H3 tail structure. **a** Interactions of the R17 backbone with the backbones of the acetylated K14 and Q19. **b** Side-chain interactions of R17 with the backbones of A25 and G33. **c** Stabilization of the two loops on top of each other via A25, P16, G34, and T11. **d** Stabilization of the bottom loop through hydrogen bonding between the backbones of Q19 and the acetylated K9, and between A21 and A7. **e** Position and orientation of the acetylated K9 and K14 side-chains (*red*). **f** Position and orientation of the trimethylated K4 and K36 (*blue*). Molecular graphics were created with YASARA (www.yasara.org) and POVRay (www.povray.org)

Figure 4 shows important interactions within the active H3 tail. The backbone amide of R17 formed a hydrogen bond with the carboxyl oxygen of the acetylated K14 (Fig. 4a). Importantly, the acetylated side-chain of K14 was not involved in any hydrogen bonds. The carboxyl oxygen of R17 was, in turn, involved in a hydrogen bond with the backbone amide of Q19.

Two of the side-chain amides of R17 were involved in two hydrogen bonds with the carboxyl oxygen of G33, while the other side-chain amide was involved in a hydrogen bond with the carboxyl oxygen of A25 (Fig. 4b). The side-chain of R17 was positioned in the middle of the loop between A25 and G33, which stabilized the loop though hydrogen bonding with both residues. The overall structure of the bulge in Fig. 4a consisted of two loops stacked on top of one another.

Figure 4c demonstrates how the stacked loops were stabilized. The carboxyl oxygen of P16, located in the bottom loop, was involved in a hydrogen bond with the backbone amide of A25, located on the top loop. The second hydrogen bond was formed between the side-chain hydroxyl oxygen of T11, located in the bottom loop, and the carboxyl oxygen of G34, located in the top loop. In contrast to the stabilization of the top loop, the bottom loop was primarily stabilized at the stem of the loop by two hydrogen bonds (Fig. 4d). The first hydrogen bond was between the carboxyl oxygen of Q19 and the backbone amide of the acetylated K9, and the second hydrogen bond was between the carboxyl oxygen of A7 and the backbone amide of A21. Neither of the side-chains of the acetylated lysines, K9 and K14, nor those of the trimethylated lysines, K4 and K36, were involved in any hydrogen-bonding interactions. Interestingly, the acetylated lysines were located directly opposite each other in the bottom loop (Fig. 4e), with both side-chains pointing upwards, and the two methylated lysines were also located directly across from one another on either end of the bulge, with their side-chains pointing outwards (Fig. 4f).

**Fig. 5a–d:**
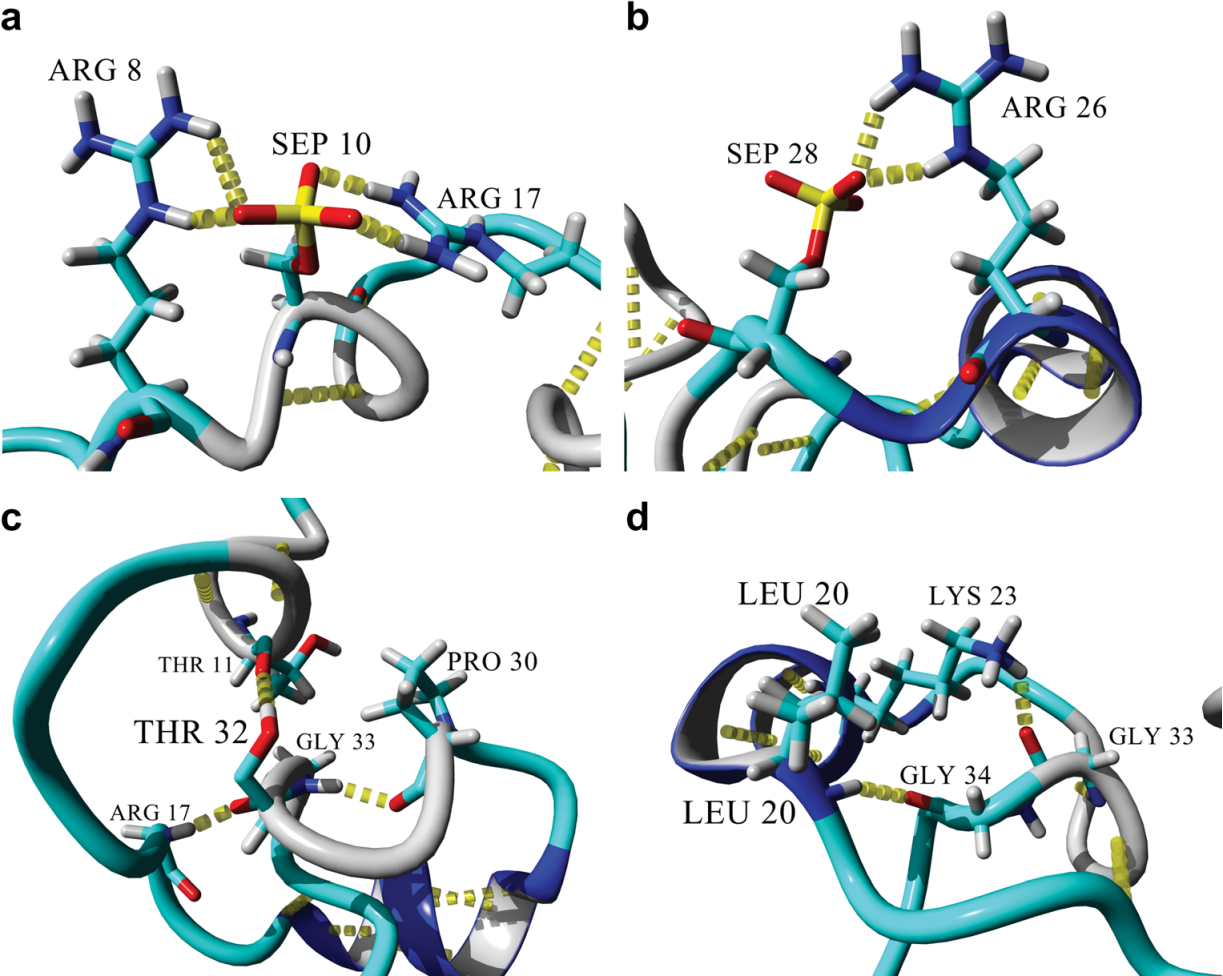
Stabilization of the inactive H3 tail structure. **a** Hydrogen bonding of the R8 and R17 side-chains with the phosphate oxygen atoms of the phosphorylated side-chain of S10. **b** Hydrogen bonding of the R26 side-chain with a phosphate oxygen atom of the phosphorylated side-chain of S28.** c** Stabilization of the hydrogen-bonded turn between G33 and P30; the backbone amide serves as hydrogen-bond donor to the backbone carbonyl oxygen of P30 and as hydrogen-bond acceptor to the backbone amide of R17. In turn, this hydrogen-bonded turn places the T32 side-chain in position to form a hydrogen bond with the backbone carbonyl oxygen of T11, further stabilizing the hydrogen-bonded turn/3_10_ helix between K9 and G13. **d** Stabilization of the N-terminal end of the α-helix between L20 and K27. The hydrogen-bonded turn between P30 and G33 positions G34 to be the hydrogen-bond acceptor at the N-terminus of the α-helix. The carbonyl oxygen of G33 also hydrogen bonds with the K23 side-chain extending from the α-helix. Molecular graphics were created with YASARA (www.yasara.org) and POVRay (www.povray.org)

In contrast, the inactive tail showed the stabilization of an α-helix between L20 and K27, a hydrogen-bonded turn/3_10_ helix between K9 and G13, and a hydrogen-bonded turn between P30 and G33 (Fig. 5a). The phosphorylated serine residues played an important role in the stabilization of the structures observed. The S10ph oxygen atoms acted as hydrogen-bond acceptors to both the guanidinium groups on the side-chains of R8 and R17, thus effectively preventing the side-chains from interacting anywhere else in the structure. The same phenomenon was seen with the side-chain of S28 and the side-chain of R26 (Fig. 5b). The hydrogen-bonded turn between G30 and G33 (Fig. 5c) was stabilized by a hydrogen bond between the two residues, while the turn was positioned in such a way that the R17 backbone amide was involved in a hydrogen bond with the carboxyl oxygen of T32. The side-chain OH group of T32 was also involved in a hydrogen bond with the carboxyl oxygen of T11. Moving towards the N-terminus of the α-helix (Fig. 5d), G34 was positioned in close proximity to L20 to enable its carboxyl oxygen to act as a hydrogen-bond acceptor for the backbone amide from L20. Additionally, the side-chain of K23 was involved in a hydrogen bond with the carboxyl oxygen of G33. The dimethylated lysines subsequently did not participate in any noticeable interactions during the formation of hydrogen bonds, with the backbone of K9 only being involved in a hydrogen bond with G12. Also, the backbone of K27 was part of the α-helix because it hydrogen-bonded with K23.

### The inactive H3 tail has a longer reach

**Fig. 6:**
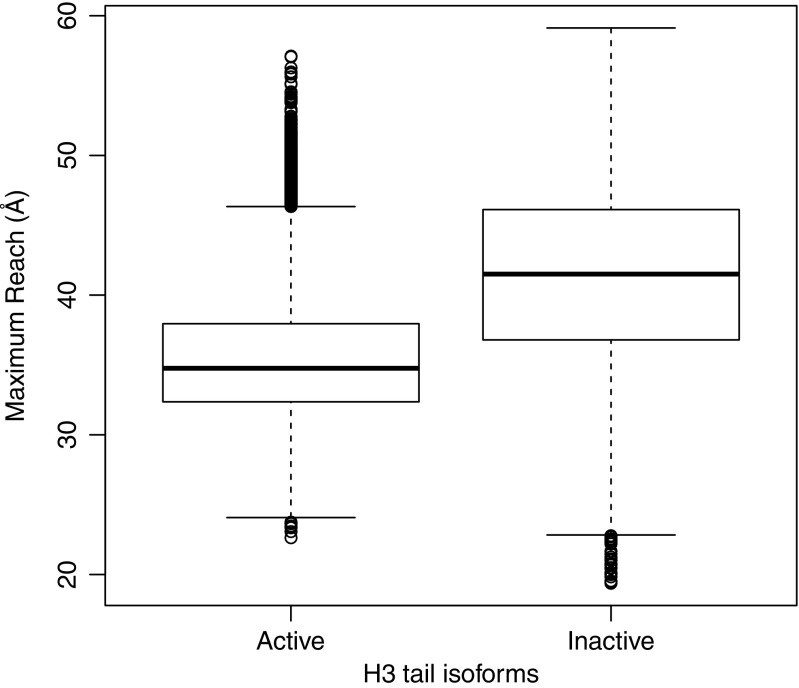
Distribution of peptide lengths of the H3 tails over the 500 ns of simulation. The* bottom*,* middle line*, and* top of each box* represent the 25th, 50th, and 75th percentiles of the tail length data, respectively. The* whiskers and dots* represent the outliers present. The inactive tail is significantly longer than the active H3 tail over the course of the simulation

From the clustered structures, it seemed that the active histone H3 tail was more compact than the inactive isoform. This difference in length may control the locations to which the tail has access within the chromatin environment, and may thus be important in explaining structural differences between transcriptionally active and silent regions of chromatin. To investigate this, we measured the length of the tail at each snapshot of the active and inactive isoforms during the respective MD runs. Figure 6 shows that the inactive H3 tail was longer than the active H3 tail, and that this difference in length was statistically significant over the entire simulation.

### Molecular docking of the H3 N-terminal tail tip isoforms to the NCP

Having established that the H3 tail showed a difference in reach between transcriptionally active and transcriptionally inactive states, the question became whether this could have any consequence in the context of chromatin structure. Since the N-terminal tails of the core histones have been shown to be bound to the NCP at low ionic strengths [45, 46], it appeared likely that the difference in length might dictate the accessibility of different binding sites.

Given the basic character of the H3 tail, the NCP contains two prominent negatively charged candidates for binding. The first is the acidic patch on the surface of the NCP, formed by aspartic acid and glutamic acid residues from histone H2A and histone H2B. This patch was shown to play a role in the regulation of chromatin compaction [47], to act as binding target for Kaposi’s sarcoma herpes virus latency associated nuclear antigen (KSHV–LANA) [26], and was also shown to bind to the N-terminal tail of histone H4 in a crystal structure of the NCP [3]. The second binding region is the two DNA gyres wrapped around the octamer and the linker DNA at the NCP termini. Many proteins recognize and bind to DNA [48, 49], and it was shown that some lysine residues in the histone H4 tail interacted with nucleosomal DNA [50].

Thus, to place the structures of the histone H3 N-terminal tail isoforms obtained from the MD simulations in the context of chromatin structure, we also need to investigate whether and where these structures bind in the NCP, and what influence the structure and modifications may have on this possible binding. Since the tail is attached to the NCP, it enforces a limit to the residues that are likely to be able to interact with the tail or other NCPs in its immediate environment. We therefore decided to investigate the binding of only the N-terminal tip (residues 1–15), and re-clustered the MD trajectories obtained according to the calculated RMSD for only the first 15 α-carbons in order to get the most represented tip structures. The unmodified trajectory yielded three clusters, while the other three trajectories only yielded one clustered structure each (Fig. S7 in the ESM).

To be confident that Autodock could provide a reliable binding configuration, we re-docked the LANA peptide [26] to the nucleosome surface, using our docking method. Autodock could indeed faithfully re-dock the peptide to the nucleosome surface in a position very similar to that seen in the co-crystal structure (Fig. S8 in the ESM).

We thus continued with our grid-based molecular docking approach to sample 3,600 possible binding positions across the NCP surface for each of the clustered structures.

**Fig. 7a–c:**
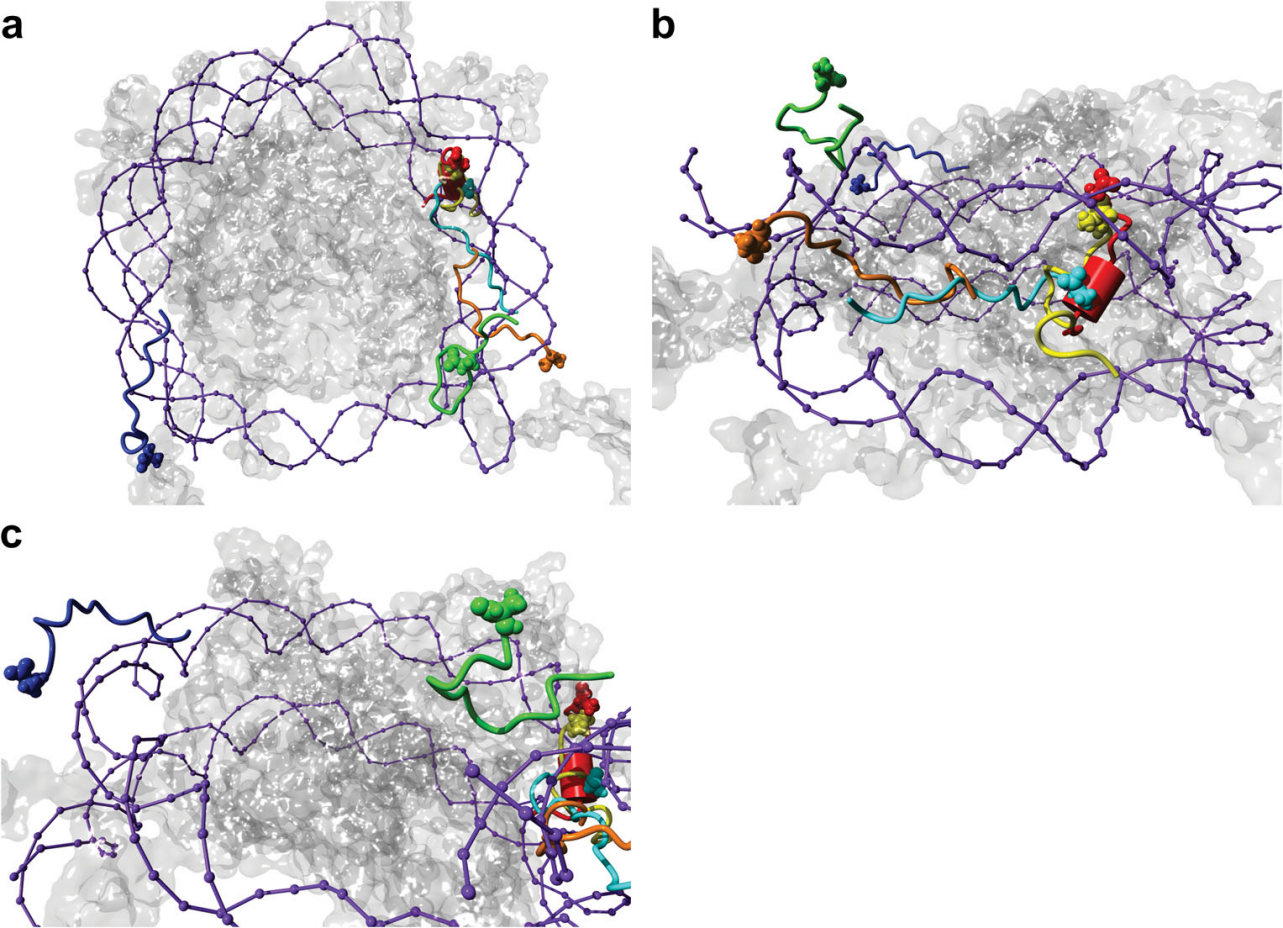
Best docking positions of cluster i of the unmodified H3 tail (*orange*), cluster ii of the unmodified H3 tail (*yellow*), cluster iii of the unmodified H3 tail (*red*), cluster i of the hyperacetylated H3 tail (*green*), cluster i of the active H3 tail (*cyan*), and cluster i of the inactive H3 tail tip (*blue*) with the nucleosome (octamer is* transparent* and DNA is indicated in* purple*). **a** The binding positions from the top of the nucleosome. **b** The binding positions from the side of the nucleosome, with every tail tip except the inactive tip in view. **c** Binding position from the side of the nucleosome, with the inactive tail tip in view. Binding energy values are highlighted in Table 2. Molecular graphics were created with YASARA (www.yasara.org) and POVRay (www.povray.org)

Figure 7 shows the top-ranked docking poses obtained for each tip structure ranked by the binding energy generated by Autodock (Table 2). The best docking poses for all of the isoforms were found between the location where the H3 and H2B tails exit the NCP, and showed distinct differences in docking between isoforms. The unmodified structures showed the highest binding scores, followed by active, inactive, and finally the hyperacetylated structures (Table 2).

**Table 2:** Best docking poses for each tip structure in each of the grid cells covering the NCP; highlighted cells indicate the best docking pose for each tip structure

	Grid cells and binding energy (kcal/mol)^a^								
	A1	A2	A3	B1	B2	B3	C1	C2	C3
Unmodified A I	3.32	3.34	4.80	2.70	3.93	7.74	5.00	4.99	8.49
Unmodified A II	3.97	4.63	7.02	3.38	3.88	8.10	5.62	4.41	6.78
Unmodified A III	4.35	3.86	9.33	3.81	4.05	9.27	4.36	3.75	6.91
Hyperacetylated B I	−2.55	−2.21	−0.24	−3.51	−3.88	−1.02	−2.80	−3.28	0.28
Active C I	0.89	0.97	1.32	−1.43	−0.79	1.93	−0.26	−0.12	1.54
Inactive D I	−0.70	-0.55	0.75	−0.85	−1.00	0.69	1.48	−0.81	−0.20

^a^ YASARA reports binding energy as the energy obtained from binding, as opposed to Autodock, which reports the energy released by binding and thus negative values; here we use YASARA’s convention.

We studied all possible contacts that could be made between each highlighted tip structure in Table 2 and the NCP, and identified all possible hydrophobic contacts and hydrogen bonds formed between the tips and the NCP (Tables S3a–S3d in the ESM). Next we focused on the best binding positions of the active and the inactive H3 tips.

### The active tip binds parallel with the DNA gyres between the exit points of histone H3 and histone H2B

The active tip was docked between the exit points of histone H3 (molecule A) and histone H2B (molecule H), and positioned vertically towards the top DNA gyre (Fig. S9a in the ESM), though there was a loop in the tip structure that pointed more towards the intersection point of the α2 α-helices from H2A, H2B, and H3 (Fig. S9b in the ESM). Interestingly, the favored position of the tip spanned two major grooves and one minor groove. The N-terminal was positioned at the side of the major groove, and was anchored by both the side-chains of R2 and the trimethylated K4 involved in a series of hydrophobic interactions with the nucleotides forming the wall of the major groove (Fig. S10 in the ESM).

The active tip displayed minimal hydrogen bonding with the DNA gyres, and was dominated by hydrophobic interactions (Fig. S10 in the ESM). The acetylated K9 side-chain was found to fit into a hydrophobic pocket formed by S52 and K54 of histone H2B, the G-56 nucleotide, and I79 and P80 of histone H2A at the position where the N-terminal ends of the α2 α-helix of H2B and the α3 α-helix of H2A met (Fig. S11 in the ESM). T11 also interacted within the hydrophobic pocket formed by S57 and K54 of H2B and Y100 of H4 (Fig. S12 in the ESM).

### The inactive tip does not bind near the exit point of histone H3 on the octamer

The inactive tip, on the other hand, did not bind near the exit point of the histone H3 A chain. Instead, it preferred to bind on top of the DNA, above the exit of the other histone H3 molecule (chain E), opposite the binding site of the other tip structures (see Fig. S13 in the ESM). Interestingly, the dimethylated K9 and phosphorylated S10 were located on a bulge with their modified side-chains pointing away from the binding site, suggesting that these modified residues would still be accessible to interact with chromatin reader proteins. With the exception of a solitary contact with R69 of histone H3, hydrogen-bonded to the same nucleotide backbone as A1, the tip showed no contacts or interactions with the octamer whatsoever (Fig. S14 in the ESM). Instead, the tip was clamped over a minor groove of DNA, with the N-terminus pointing towards the octamer and A1, R2, T6, and R8 hydrogen-bonded with the DNA backbone (see Fig. S15a in the ESM). Multiple hydrophobic interactions were also observed between several tip residues and the DNA (see Fig. S15b in the ESM).

### The tail tips behave according to the docking results after a 10-ns MD simulation

Given the flexibility of the peptides, we conducted a short 10-ns MD simulation on the tips to assess the stability of the bound conformations. Because we only used a portion of the tail to probe the nucleosomal surface, some additional flexibility could be expected near the N-termini of the tips. Indeed, Fig. S16 in the ESM shows that tips remained bound near their docked positions, with the most structural variation found at the N-termini of the tips. The inactive tail tip showed the most deviation from its docked position, which was in line with the binding energy result. The α-C RMSD for the tips during the short simulation (Fig. S17 in the ESM) loosely corresponded with the binding energy results, with the unmodified tail tips showing a smaller change in RMSD than the modified tail tips.

### The active tail can only form intranucleosomal contacts, while the inactive tail can form both intra- and internucleosomal contacts

**Fig. 8a–d:**
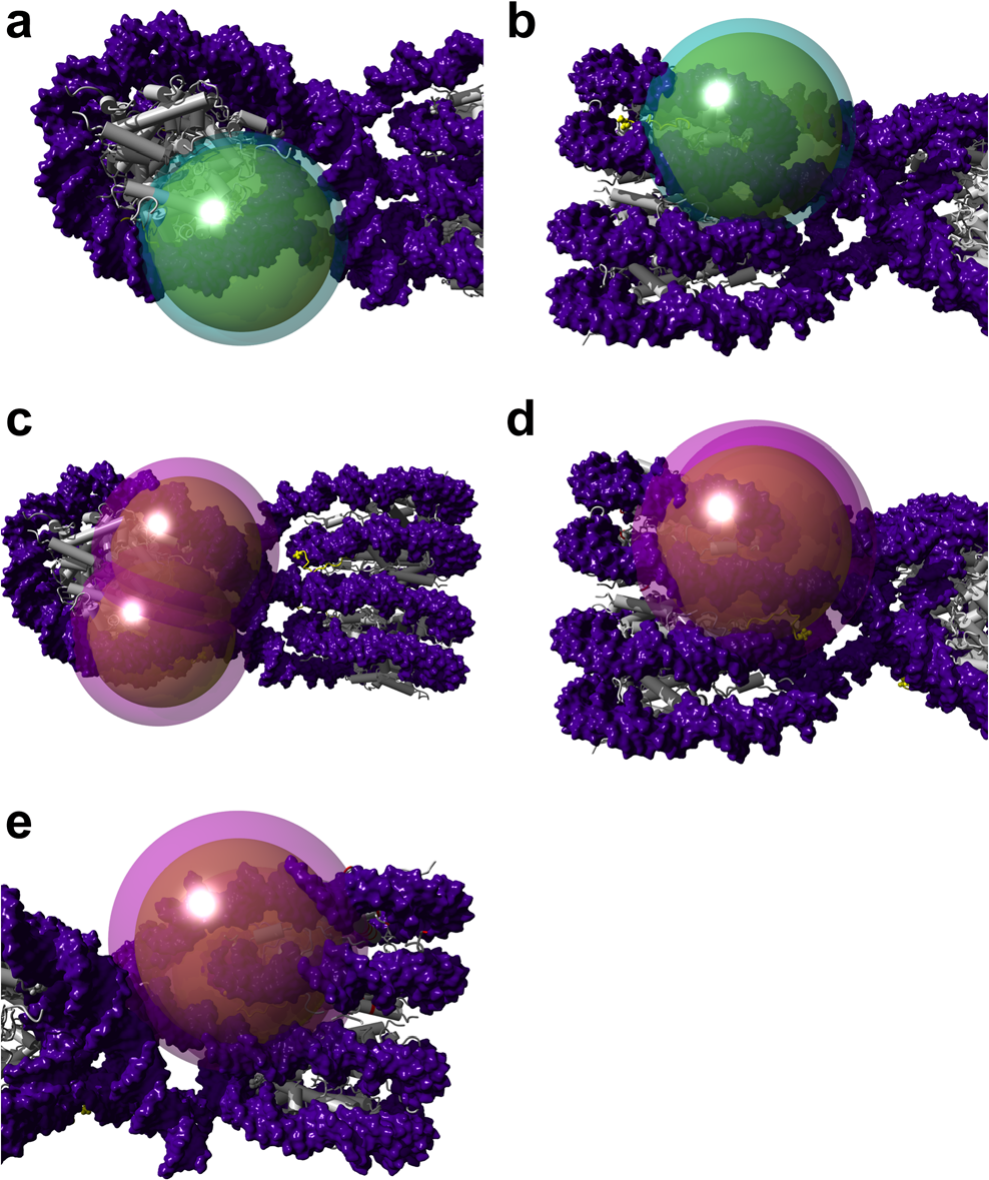
Model of the active and inactive tails within compact chromatin. **a** The tail reach of the active tail during MD simulations. **b** The active tail can only reach the binding position of its tip in its parental nucleosome. **c** The tail reach of the inactive tail during MD simulations, as observed from above, indicates that it too cannot reach the adjacent nucleosomal stack. **d** The inactive tail can reach its binding position within the nucleosome below it, as well as **e** in its parental nucleosome. DNA is indicated in* purple*, the octamer in* gray*, and the binding positions of the tips are indicated in* yellow*. Molecular graphics were created with YASARA (www.yasara.org) and POVRay (www.povray.org)

To place the MD simulations and the docking data into the context of chromatin, we used the structure of the tetranucleosome (1ZBB) to overlay the best binding positions of both the active and inactive tails onto the individual nucleosomes in the structure. We constructed dual-layered spheres to represent the reaches of the active and inactive tails according to the reach data in Fig. 6. Figures 8a and b show that the active tail can only contact its best binding position within its parental nucleosome. The inactive tail, in contrast, is able to contact its best binding position in its parental nucleosome as well as the binding position in the nucleosome below (or above) in the same nucleosomal stack (Fig. 8c–e). For both tails, the reach precludes contact with the nucleosomes on the adjacent stack (Fig. 8), and the tails are also not able to contact the acidic path formed by histone H2A/H2B (Fig. S18 in the ESM).

## Discussion

In this study, we have demonstrated, using MD simulations, that the free, unmodified histone H3 N-terminal tail has the propensity to form a structure composed of two α-helices. The first, which we called the tip helix, is formed between T3 and S10, and the second, which we called the middle helix, is formed between L20 and R26. The tip helix was more stable than the middle helix during our simulations. A similar propensity for α-helical content in various shorter lengths of the H3 N-terminal tails was reported using MD [17, 18], while two α-helices, one between K4 and K9 and a second between K14 and R26, have been observed in the N-terminal tail of H3 during a MD simulation of the nucleosome [21]. CD experiments indicated that the H3 and H4 tails contributed to the α-helical character of the nucleosome [15, 16], and an X-ray crystal structure of the double PHD finger domain of MOZ/MYST3 in complex with three histone H3 tail isomers, including an unmodified isomer, showed an α-helical conformation between K4 and T11 in the H3 tail [51].

Both the hyperacetylated tail and active tail abolished the two α-helices observed in the unmodified tail and produced remarkably similar structures. This is an interesting finding, given that both tails are associated with permissive chromatin structures [27, 52]. In the hyperacetylated tail, we observed β-bridge content as well as a preference for hydrogen-bonded turns. In contrast, in the active tail, none of the modified side-chains (both acetylated and methylated) participated in direct interactions within the tail itself. We also observed that R17 formed the nucleus of the hydrogen-bonding network stabilizing the two loops stacked on top of one another in the active structure.

The inactive tail only reinstated the middle helix to the peptide structure, but it did so effectively, by stabilizing the most consistent secondary structure formed during our study. Eberlin and coworkers confirmed, using cryo-EM and biomolecular modeling, that H3 tails containing the modifications found in our inactive tail lead to a conformational change between mitosis and meiosis, and that an antibody could be raised that recognized the unique structure of this inactive tail [28]. This supports the increased stability of the inactive structure that we found in our simulations.

Furthermore, we observed that the different structures observed for the inactive tail were affected by the immobilization of the R8, R17, and R26 side-chains via the phosphorylated S10 and S28. This allowed for the establishment of an elaborate hydrogen-bonding network involving T11, the R17 backbone, K23, P30, T32, and G33, which placed G34 in a position where it could accept the hydrogen bond from the L20 backbone amine to effectively cap the N-terminal of the α-helix and thus stabilize the structure. Similar interactions of phosphorylated side-chains with arginine side-chains have been observed in both MD simulations [53] and NMR experiments [54] before, thus suggesting that that these interactions were not artifactual. In comparison with the active tail structure, the side-chain of R17 played a pivotal role in essentially preventing the contacts necessary to form the hydrogen-bonding network observed in the inactive tail.

It is important to note that except for the direct interaction of the phosphorylated serine residues in the inactive tail and select acetylated side-chains in the hyperacetylated tail, none of the other modified residues participated in any direct interaction within the tail structures. Indeed, in both the active and inactive tails, the modified lysine side-chains tended to protrude into the solvent. This observation is consistent with the histone code hypothesis [10]. Many effector proteins are known to bind to the modifications used in the study. For example, the TAF3-PHD finger recognizes and binds K4Me3 [55, 56], SIRT6 deacetylates K9ac [57], LSD1 demethylates K9Me2 [58], MKP-1 dephosphorylates S10, and PHF8 demethylates both K9Me1/2 and K27Me2 [59]. Also, given its prominence in the structures observed in this study, R17, while unmodified here, is also the target of methylation by CARM1 [60], while deamination of R17 (together with R2 and R26) by PAD4 [61] also plays a role in the development of multiple sclerosis [62].

We have, for the first time, produced evidence that different PTM patterns can potentially produce different H3 tail structures. What functional relevance may this have in terms of chromatin compaction? We noticed that the top inactive structure seemed to have a longer reach than the top active tail structure. Indeed, we showed this to be statistically significant in our simulations. Since this finding could be significant in chromatin compaction, we used the basic unit of the cryo-EM structure of a compacted chromatin fiber [63], the tetranucleosome, and identified—given the average reach distances during our MD simulations—positions that the active and inactive H3 tails could potentially reach within the condensed chromatin fiber. We found that the H3 tail could not reach the nucleosome(s) in the adjacent nucleosome stack. It was, however, possible for the tails to make contact with the nucleosome(s) above or below it in the same stack. These regions included the nucleosomal DNA, the linker DNA between the stacks, and also potentially histone H1, given the position of H1 at the entry/exit point of the linker DNA [64]. The acidic patch formed between H2A/H2B has been shown to be an important binding position on the nucleosome surface, with both the histone H4 N-terminal tail [3] and KSHV-LANA [26] binding to it [47]. However, based on the most common reach distances of the H3 tails, the longer H3 tail could not reach this potential binding surface.

We probed the entire nucleosome surface and DNA for possible binding positions using the 15-residue tip of the H3 isoforms with molecular docking. We confirmed that the H3 tip showed little affinity for the acidic patch, and instead we identified two other potential binding sites: the first to the side of the nucleosome between the octamer and DNA, and the second site on top of the DNA strands on both sides of the dyad. Existing protein–DNA crosslinking studies performed on the mono-, di- and oligonucleosomal model systems confirmed that the H3 tail contacted the DNA in the nucleosome [65].

In terms of binding energies, the unmodified tail tips had a strong affinity for the nucleosome at a site close to the exiting H3 tail exit point, between the side of the octamer and the DNA. We speculate that overall structure, the steric hindrance introduced by PTMs, and the change in electrostatic charge of the tails contributed to the calculated binding energies. The unmodified tail containing the α-helix produced the most favorable binding position, exhibiting unique interactions due to the positioning of residues by the α-helix, while the tail with the most PTMs and lowest net charge, the hyperacetylated H3 tail, produced the most unfavorable binding energies. This supports the finding by Mutskov and coworkers that the tail–DNA interactions are mostly electrostatic in nature [66].

While the hyperacetylated tail tip exhibited an unfavorable binding energy, it still retained a total number of DNA contacts comparable to the unmodified tip, and this agreed with experimental evidence that acetylated histone tails show no difference in their tail–DNA contacts from unmodified histone tails at 100–150 mM NaCl, but start to lose contacts at a higher salt concentration when the NaCl concentration is increased [66]. In light of biophysical evidence [22, 23, 25, 67] which indicated that the terminal nucleosomal DNA becomes more mobile upon hyperacetylation of the H3 and H4 tails, we speculate that the tail may remain attached to the DNA, albeit weakly, with the decrease in tail reach contributing to lifting the terminal DNA off the octamer surface.

To understand the possible structural role of the H3 tail in chromatin, we superimposed the binding positions of the both the active and inactive tail tip onto the model of the tetranucleosome. Together with the MD reach data already inserted into the model, we confirmed that the inactive tail was capable of reaching two potential binding sites per H3 molecule: one on its parental nucleosome and another on a nucleosome above/below it in the same nucleosome stack. The active tail could only reach the position in its parental nucleosome. It is plausible that open chromatin would not have H1 bound [12], while in compact chromatin, H1 would be present [68]. Thus, the observation that an inactive H3 tail can have more than one binding position within our model is consistent with evidence that the binding of the linker histone caused rearrangement in tail–DNA contacts [69].

Furthermore, it has been shown that in an extended, uncompact array, the histone H3 tail makes predominantly intranucleosomal contacts, which agrees with the binding position we found in our docking experiments. Likewise, the binding position of the inactive tail on the DNA agrees with the finding that the H3 tail–DNA interactions are reorganized to form predominantly internucleosomal interactions, with 20% of these being interarray and 80% being intra-array, during salt-dependent folding [70, 71]. The weaker binding energy observed with the inactive tail is also supported by a study by Sauvé and coworkers, who observed a weakening of the interaction between the H3 tail and the DNA during mitotic chromatin condensation [72].

Thus, in accordance with experimental data, we propose that distinct PTM patterns change the structure of the H3 N-terminal tail, and that this either increases its reach in the case of chromatin compaction or decreases the reach of the tail in chromatin decondensation. The tail in compact chromatin can therefore participate in more internucleosomal interactions, leading to more compact chromatin structures, while the tail in decondensed chromatin can predominantly form intranucleosomal interactions, which leads to a more open chromatin structure. The binding positions towards the side of the nucleosome and the observation that the modified lysine side-chains protrude into the solvent with no direct role with in the peptide structure also make it possible for effector proteins to bind. Curiously, the absence of structure in both the active and inactive tail tips is also in accordance with resolved structures of modified H3 tail tips bound by effector proteins, which show a random coil structure for the tail tips [73, 74]

In conclusion, we have shown in this study that the unmodified H3 tail stabilized two α-helices, that the introduction of PTMs changed the structures that were stabilized, and that a combination of the structural change induced and the changes in the physicochemical properties of the N-terminal tips of these tails effected a change in the binding properties of the tail tip to the nucleosome in a tetranucleosomal model. By integrating our data with existing biochemical and biophysical data, we are able to propose the basis for a model for chromatin compaction mediated by PTMs.

## Electronic supplementary material

Below is the link to the electronic supplementary material.

ESM 1 (DOCX 15341 kb)
