# Supplementary material for: The effect of epigenetic modifications on the secondary structures and possible binding positions of the N-terminal tail of histone H3 in the nucleosome: a computational study
Source: J Mol Model. 2017 Mar 28;23(4):137. doi: 10.1007/s00894-017-3308-x (PMC5391383; doi:10.1007/s00894-017-3308-x)
Supplement: Supplementary file 1 — (DOCX 15341 kb) [file 894_2017_3308_MOESM1_ESM.docx]

**Supplementary Table S1.** Internet – based algorithms for secondary structure prediction, used with default settings, used in this study

| Name | Description | URL | Reference |
| --- | --- | --- | --- |
| CFSSP | Chou & Fasman Secondary Structure Prediction Server | <http://www.biogem.org/tool/chou-fasman/> | (1) |
| Jpred | A consensus method for protein secondary structure prediction at University of Dundee | <http://www.compbio.dundee.ac.uk/~www-jpred/> | (2) |
| JUFO | Protein secondary structure prediction from sequence (neural network) | <http://meilerlab.org/index.php/servers/show?s_id=8> | (3) |
| NSurfP | Protein Surface Accessibility and Secondary Structure Predictions | <http://www.cbs.dtu.dk/services/NetSurfP/> | (4) |
| Porter | Protein Secondary Structure Prediction at University College Dublin | <http://distill.ucd.ie/porter/> | (5-7) |
| PredictProtein | Sequence Analysis, Structure and Function Prediction | <http://www.predictprotein.org/> | (8;9) |
| Prof | Cascaded Multiple Classifiers for Secondary Structure Prediction | <http://www.aber.ac.uk/~phiwww/prof/> | (10) |
| Scratch Protein Predictor | SSpro and SSpro8 | <http://scratch.proteomics.ics.uci.edu/index.html> | (11;12) |
| NPS@ | Secondary Structure Consensus Prediction | <http://npsa-pbil.ibcp.fr/cgi-bin/npsa_automat.pl?page=/NPSA/npsa_seccons.html> | (13) |
| PSIPRED | Protein secondary structure prediction based on position-specific scoring matrices | <http://bioinf.cs.ucl.ac.uk/psipred/> | (14) |

**Supplementary Table S2.** PDB source structures of modified residues used in MD simulations. Three letter abbreviations in brackets are indicative of the original names for the residues in the structure files, which were changed for this study

| **Post Translational Modification** | **Source PDB Code** | **Chain in PDB structure** | **Residue Number in PDB structure** | **Residue three letter abbreviation** | **Reference** |
| --- | --- | --- | --- | --- | --- |
| Mono-methylated Lysine | 3HNA | P | 9 | M1L (MLZ) | (15) |
| Di – methylated Lysine* | 2KVM | B | 27 | M2L (MLY) | (16) |
| Tri – methylated Lysine* | 2L11 | B | 9 | M3L | (17) |
| Acetylated Lysine* | 2KWJ | B | 14 | ALY | (18) |
| Phosphorylated Serine | 2C1N | C | 10 | SEP | (19) |
|  |  |  |  |  |  |

* For NMR structures, the first structure in the file was selected.

**Supplementary Figure S1.** The docking grid covering the nucleosome surface and DNA. I shows the non – overlapping grid cells and II shows the overlapping grid cells. *Molecular graphics created with YASARA (www.yasara.org) and POVRay (www.povray.org)*

**Supplementary Figure S2.** Secondary structure prediction of the 43-residue N – terminal tail of histone H3 using the algorithms indicated in Supplementary Table S1. The highlighted residues indicate residues which contained PTMs in this study.


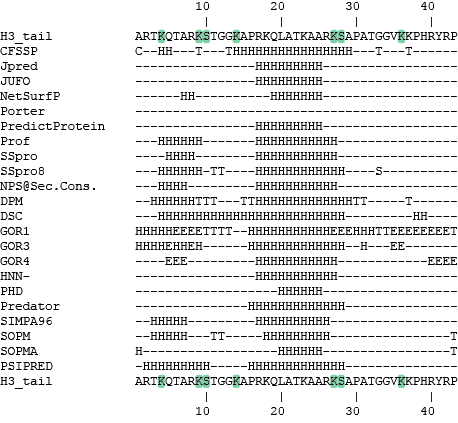


**Supplementary Figure S3.** Evolution of the secondary structure content of (A) the Ala_43_ H3 tail and (B) the Gly_43_ H3 tail over the course of a 500 ns all-atom MD simulation. Random Coil and other elements are indicated in white, β – strands in purple, β-bridges in black, hydrogen bonded turns in grey, 3^10^ – helices in yellow and α – helices in blue.

.
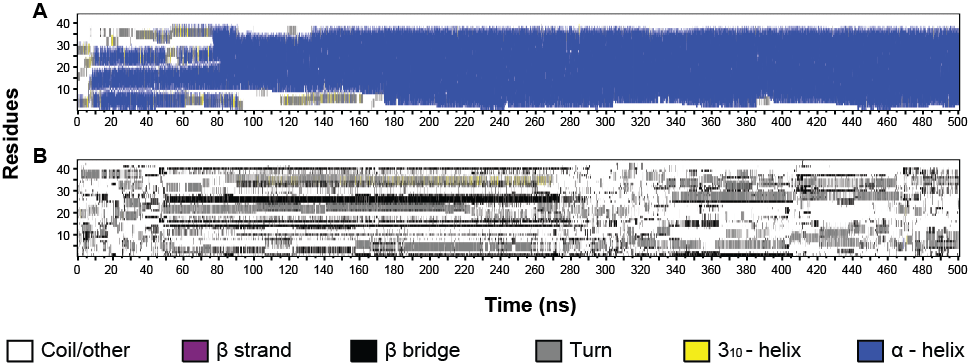


**Supplementary Figure S4.** The fractional distribution in percentage of simulation time of secondary structures at each residue over the 500 ns all-atom MD simulation of (A) the Ala_43_ H3 tail and (B) the Gly_43_ H3 tail. Random Coil and other elements are indicated in white, β – strands in purple, β-bridges in black, hydrogen bonded turns in grey, 3^10^ – helices in yellow and α – helices in blue.


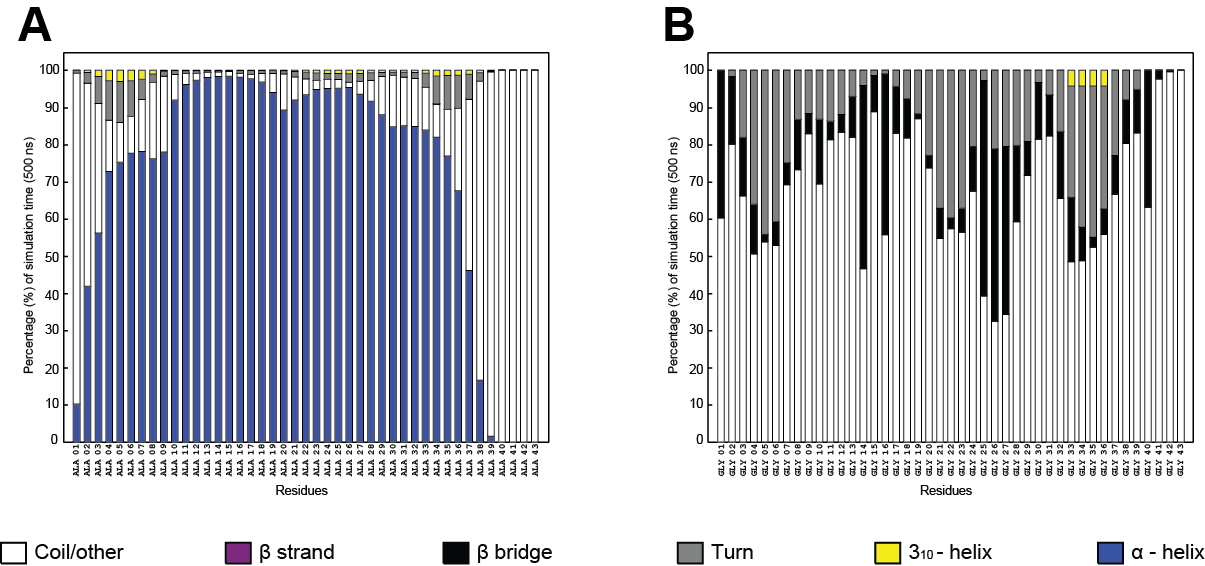


**Supplementary Figure S5.** Representative structures of the top most populated clusters found for the unmodified H3 tail (A), the hyper-acetylated H3 tail (B), the active H3 tail (C) and the inactive H3 tail (D). The lower-case roman numerals indicate the rank of the cluster. The asterisk indicates the C-terminal of each structure and the time at which the structure occurs is indicated. Random Coil and other elements are indicated in cyan, β – strands in purple, β-bridges in black, hydrogen bonded turns in grey, 3^10^ – helices in yellow and α – helices in blue.

**
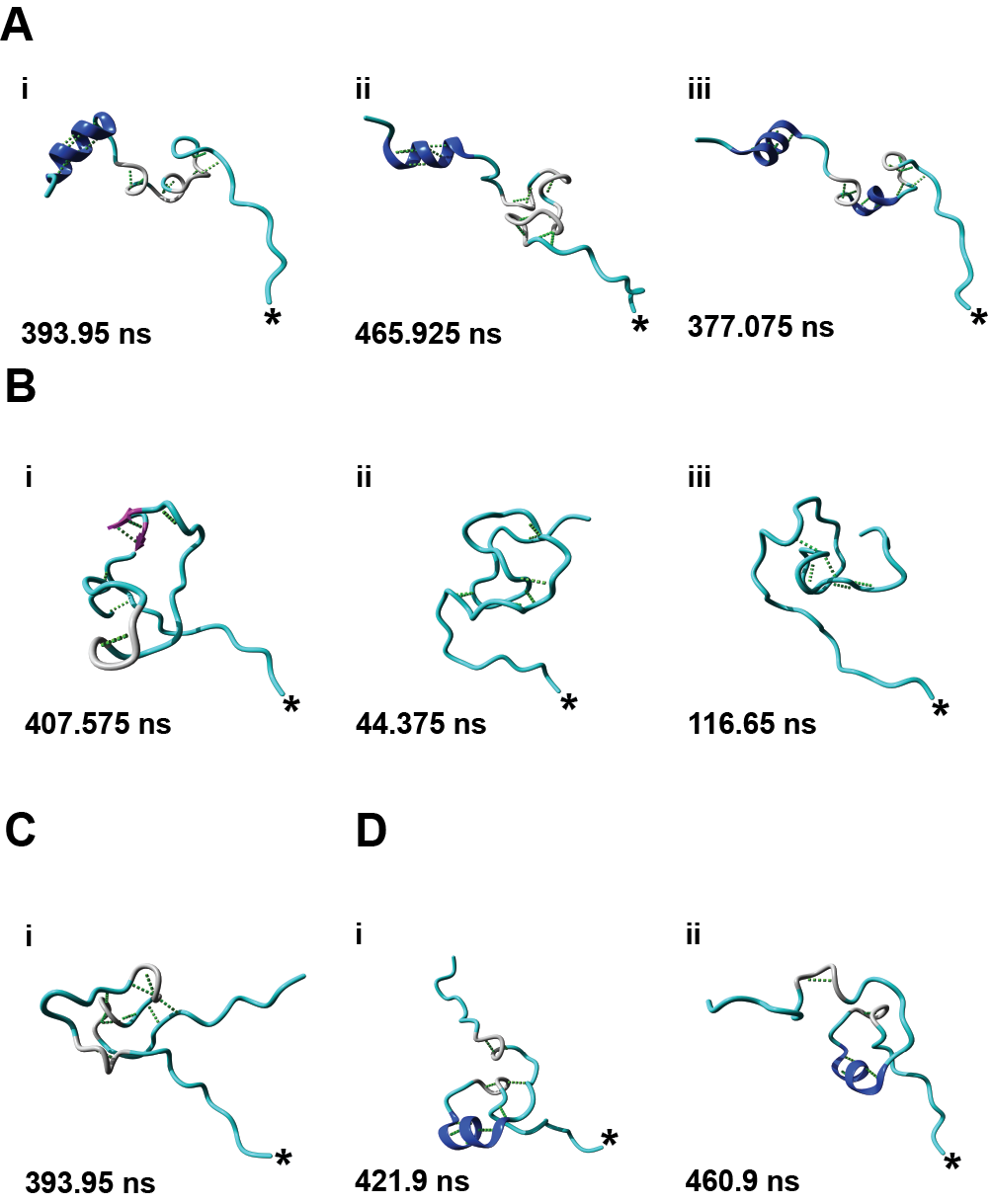
**

**Supplementary Figure S6.** Existence of hydrogen bonds found in the top clusters for the active and inactive H3 tails throughout the simulation time.

**
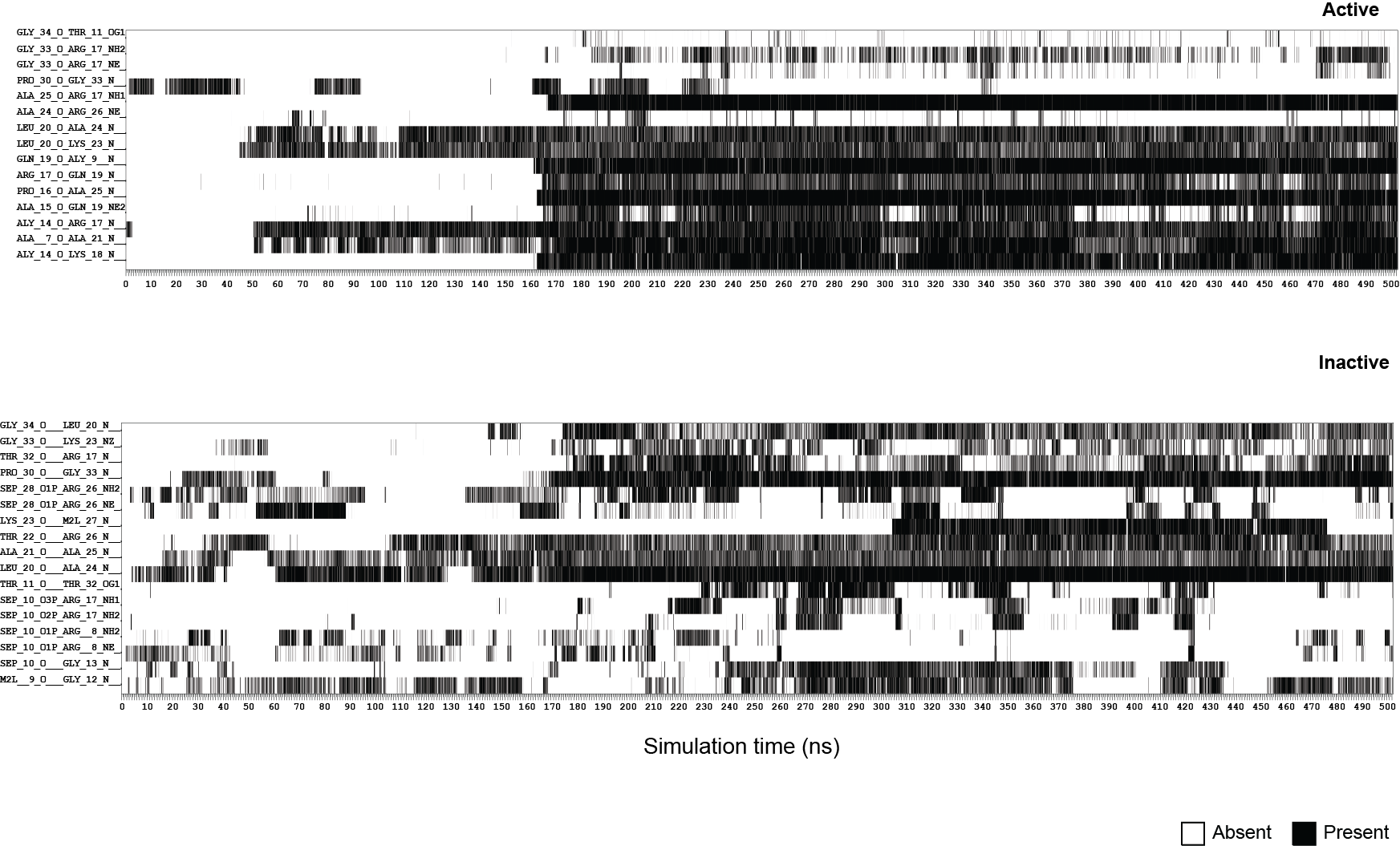
**

**Supplementary Figure S7.** Representative structures of the top most populated clusters found for the 15 – residue N-terminal tip of the unmodified H3 tail (A), the hyper-acetylated H3 tail (B), the active H3 tail (C) and the inactive H3 tail (D). Roman numerals indicate the time-order of the structures, while the values in brackets indicates the cluster rank of the structure. Random Coil and other elements are indicated in cyan, β – strands in purple, β-bridges in black, hydrogen bonded turns in grey, 3^10^ – helices in yellow and α – helices in blue


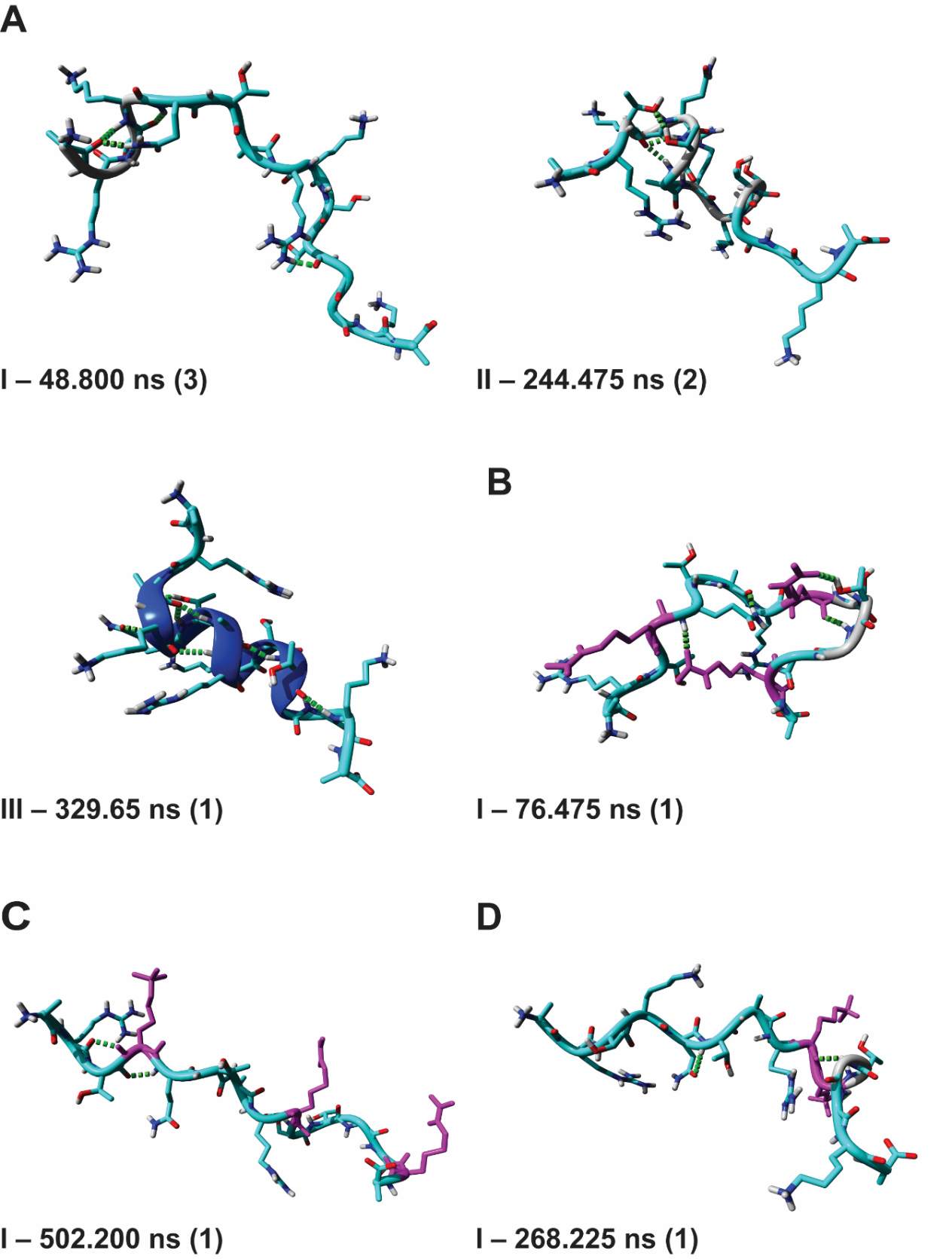


**Supplementary Figure S8.** Redocking of the KSHV – LANA peptide to the surface of the NCP. Docked structures are indicated in yellow, while the original crystal structure is indicated in magenta. Only grid cells A2, A3, B2 and B3 covered the crystal docking site

**
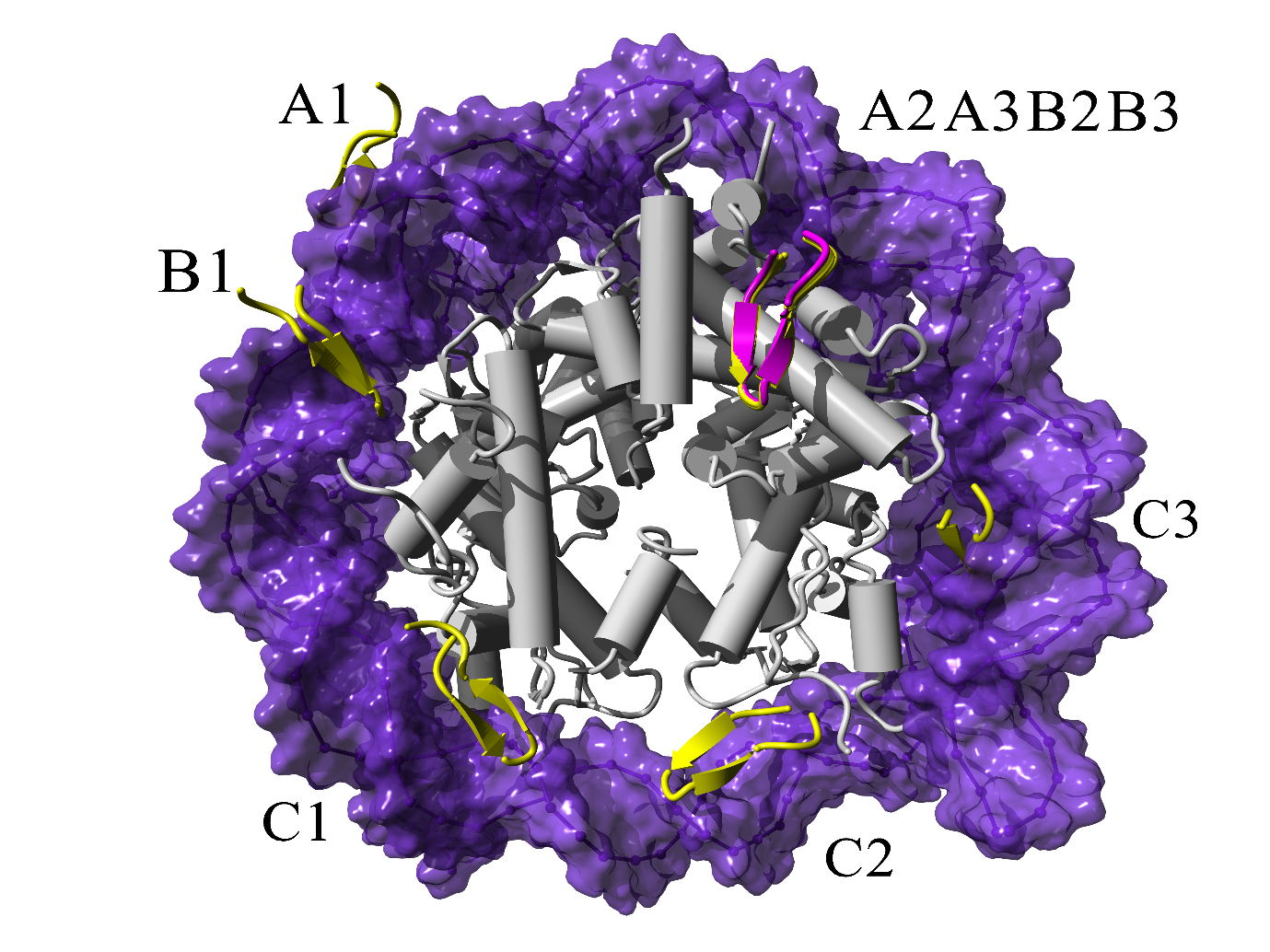
**

**Supplementary Table S3 AI i.** All contacts found between the highest ranked docking orientation of the unmodified AI tip structure and the NCP (See Figure S5 AI). Molecule names are as they are defined in the PDB structure 1KX5.

| **Tip Residue** | **Interacting Partner** | **Distance (Å)** | **Number of Contacts** | **Total Number of Contacts** |
| --- | --- | --- | --- | --- |
|  | **<Molecule Name> <Residue Name> <Residue Number>** |  |  |  |
| ALA 1 |  |  |  | 2 |
|  | J DA -57 | 3.87496 | 1 |  |
|  | J DG -56 | 3.37634 | 1 |  |
| LYS 9 |  |  |  | 54 |
|  | A VAL 46 | 3.55934 | 1 |  |
|  | A ARG 53 | 3.95008 | 1 |  |
|  | J DT -66 | 2.61859 | 8 |  |
|  | J DA -67 | 2.84448 | 9 |  |
|  | A ARG 49 | 1.85262 | 35 |  |
| SER 10 |  |  |  | 54 |
|  | J DT -68 | 3.79859 | 1 |  |
|  | J DA -67 | 2.33295 | 20 |  |
|  | J DT -66 | 2.08941 | 33 |  |
| THR 11 |  |  |  | 67 |
|  | I DG 65 | 3.496 | 1 |  |
|  | J DC -64 | 3.43769 | 1 |  |
|  | I DG 64 | 3.00726 | 4 |  |
|  | J DT -66 | 2.01015 | 11 |  |
|  | J DC -65 | 2.30305 | 16 |  |
|  | I DT 63 | 1.15793 | 34 |  |
| THR 6 |  |  |  | 55 |
|  | I DG -15 | 3.58494 | 2 |  |
|  | A ARG 53 | 1.77282 | 26 |  |
|  | I DC -14 | 1.78346 | 27 |  |
|  |  |  |  |  |
| GLY 13 |  |  |  | 43 |
|  | I DT 63 | 3.71516 | 2 |  |
|  | I DG 65 | 2.10041 | 18 |  |
|  | I DG 64 | 2.44116 | 23 |  |
| THR 3 |  |  |  | 25 |
|  | A GLU 59 | 3.26806 | 4 |  |
|  | I DA 60 | 2.86402 | 10 |  |
|  | I DG 61 | 1.85345 | 11 |  |
| GLY 12 |  |  |  | 4 |
|  | J DT -66 | 3.05368 | 4 |  |
|  |  |  |  |  |
|  |  |  |  |  |
|  |  |  |  |  |
| LYS 14 |  |  |  | 109 |
|  | J DA -67 | 2.70099 | 4 |  |
|  | I DG 64 | 3.41442 | 7 |  |
|  | I DT 67 | 2.12492 | 25 |  |
|  | I DA 66 | 1.98702 | 35 |  |
|  | I DG 65 | 1.92618 | 38 |  |
| LYS 4 |  |  |  | 64 |
|  | A GLU 59 | 3.0736 | 5 |  |
|  | G GLN 104 | 1.86854 | 26 |  |
|  | H LYS 54 | 2.1689 | 33 |  |
| GLN 5 |  |  |  | 12 |
|  | I DG 61 | 2.72133 | 6 |  |
|  | A GLU 59 | 3.59732 | 6 |  |
| ARG 2 |  |  |  | 138 |
|  | J DA -57 | 3.16661 | 9 |  |
|  | J DG -59 | 2.53886 | 19 |  |
|  | J DC -58 | 2.46291 | 28 |  |
|  | I DG 61 | 2.17005 | 30 |  |
|  | I DA 60 | 1.46859 | 52 |  |
| ARG 8 |  |  |  | 26 |
|  | I DG 62 | 2.68937 | 12 |  |
|  | I DT 63 | 2.20001 | 14 |  |
| ALA 7 |  |  |  | 23 |
|  | A ARG 53 | 1.7647 | 23 |  |
|  |  |  |  |  |

**Supplementary Table S3 AI ii.** Hydrophobic contacts found between the highest ranked docking orientation of the unmodified AI tip structure and the NCP (See Figure S5 AI). Molecule names are as they are defined in the PDB structure 1KX5.

|  |  |  |  |  |
| --- | --- | --- | --- | --- |
| **Tip Residue** | **Interacting Partner** | **Distance (Å)** | **Number of Contacts** | **Strength of interaction** |
|  | **<Molecule Name> <Residue Name> <Residue Number>** |  |  |  |
| LYS 4 |  |  |  |  |
|  | G GLN 104 | 4.40102 | 1 | 0.884055793 |
|  | H LYS 54 | 4.01453 | 2 | 1.532227755 |
| SER 10 |  |  |  |  |
|  | J DA -67 | 4.71987 | 1 | 0.757023931 |
|  | J DT -66 | 4.53187 | 1 | 0.820284069 |
| THR 11 |  |  |  |  |
|  | I DT 63 | 4.30787 | 1 | 0.912586451 |
| GLY 13 |  |  |  |  |
|  | I DG 64 | 4.90342 | 1 | 0.683896422 |
| LYS 14 |  |  |  |  |
|  | I DT 67 | 4.51488 | 1 | 0.874047995 |
|  | I DG 65 | 4.48674 | 2 | 1.582254887 |
| LYS 9 |  |  |  |  |
|  | A ARG 49 | 4.43059 | 2 | 1.712864518 |
| ARG 2 |  |  |  |  |
|  | I DA 60 | 4.14086 | 3 | 3.093673229 |
|  | I DG 61 | 4.08038 | 3 | 2.509502888 |

**Supplementary Table S3 AI iii.** Hydrogen bonds formed between the highest ranked docking orientation of the unmodified AI (See Figure S5 AI) tip structure and the NCP. Molecule names are as they are defined in the PDB structure 1KX5

| **Tip Residue** | **Interacting Partner** | **Hydrogen Bond Energy**  **(kJ/mol)** | **Role** | **Donor**  **Atom** | **Acceptor**  **Atom** |
| --- | --- | --- | --- | --- | --- |
|  | **<Molecule Name> <Residue Name> <Residue Number>** |  |  |  |  |
| ARG 2 |  |  |  |  |  |
|  | J DC -58 | 6.55 | Donor | NH1 | O4* |
| THR 3 |  |  |  |  |  |
|  | I DG 61 | 21.875 | Donor | N | O1P |
| THR 6 |  |  |  |  |  |
|  | I DC -14 | 13.3 | Donor | OG1 | O1P |
| SER 10 |  |  |  |  |  |
|  | J DA -67 | 13.35 | Donor | OG | O2P |
| LYS 14 |  |  |  |  |  |
|  | I DT 67 | 18.2 | Donor | NZ | O4 |
|  |  |  |  |  |  |

**Supplementary Table S3 AII i**. All contacts found between the highest ranked docking orientation of the unmodified AII tip structure and the NCP (See Figure S5 AII). Molecule names are as they are defined in the PDB structure 1KX5.

| **Tip Residue** | **Interacting Partner**  **<Molecule Name> <Residue Name> <Residue Number>** | **Distance (Å)** | **Number of Contacts** | **Total Number of Contacts** |
| --- | --- | --- | --- | --- |
|  |  |  |  |  |
| LYS 4 |  |  |  | 85 |
|  | I DC -21 | 3.58958 | 1 |  |
|  | J DG 21 | 1.85063 | 22 |  |
|  | J DT 20 | 2.10325 | 31 |  |
|  | J DA 22 | 1.61278 | 31 |  |
| SER 10 |  |  |  | 74 |
|  | J DA -57 | 3.9983 | 1 |  |
|  | J DA -55 | 2.40271 | 10 |  |
|  | H SER 53 | 2.13202 | 20 |  |
|  | J DG -56 | 1.57525 | 43 |  |
| THR 11 |  |  |  | 1 |
|  | J DG -56 | 3.84307 | 1 |  |
| GLY 13 |  |  |  | 1 |
|  | H SER 53 | 3.82955 | 1 |  |
| LYS 14 |  |  |  | 157 |
|  | I DG 48 | 3.7045 | 1 |  |
|  | I DT 50 | 2.65126 | 6 |  |
|  | H ILE 36 | 3.31828 | 8 |  |
|  | H LYS 31 | 1.77074 | 10 |  |
|  | H ALA 35 | 2.71339 | 11 |  |
|  | H GLU 32 | 1.55777 | 24 |  |
|  | H SER 33 | 1.99577 | 31 |  |
|  | I DG 49 | 1.65876 | 66 |  |
|  |  |  |  |  |
| ALA 15 |  |  |  | 20 |
|  | J DA -53 | 3.82092 | 1 |  |
|  | J DA -55 | 3.31835 | 3 |  |
|  | J DT -54 | 2.41398 | 16 |  |
| LYS 9 |  |  |  | 47 |
|  | H SER 53 | 3.12126 | 5 |  |
|  | B GLY 99 | 2.07388 | 6 |  |
|  | H SER 57 | 3.3386 | 6 |  |
|  | H LYS 54 | 2.9618 | 12 |  |
|  | B PHE 100 | 2.7317 | 18 |  |
| ARG 2 |  |  |  | 28 |
|  | I DT -26 | 3.46593 | 6 |  |
|  | I DC -25 | 1.82527 | 22 |  |
|  |  |  |  |  |
| ALA 1 |  |  |  | 72 |
|  | I DC -27 | 3.11709 | 8 |  |
|  | I DC -25 | 1.89098 | 24 |  |
|  | I DT -26 | 1.88931 | 40 |  |
| ARG 8 |  |  |  | 26 |
|  | J DA -57 | 2.13975 | 9 |  |
|  | J DG -56 | 1.98853 | 17 |  |
| GLN 5 |  |  |  | 10 |
|  | J DA -57 | 2.45048 | 10 |  |

**Supplementary Table S3 AII ii.** Hydrophobic contacts found between the highest ranked docking orientation of the unmodified AII tip structure and the NCP (See Figure S5 AII). Molecule names are as they are defined in the PDB structure 1KX5.

| **Tip Residue** | **Interacting Partner** | **Distance (Å)** | **Number of Contacts** | **Strength of interaction** |
| --- | --- | --- | --- | --- |
|  | **<Molecule Name> <Residue Name> <Residue Number>** |  |  |  |
|  |  |  |  |  |
| ALA 1 |  |  |  |  |
|  | I DC -25 | 4.12171 | 1 | 0.975691497 |
|  | I DT -26 | 4.03852 | 2 | 1.726811647 |
| LYS 9 |  |  |  |  |
|  | H LYS 54 | 4.67964 | 1 | 0.773051798 |
|  | H SER 57 | 4.78977 | 1 | 0.72917527 |
|  | B PHE 100 | 4.68108 | 2 | 1.509719849 |
| SER 10 |  |  |  |  |
|  | H SER 53 | 4.77461 | 1 | 0.735215127 |
|  | J DG -56 | 3.80341 | 2 | 1.290964127 |
| LYS 14 |  |  |  |  |
|  | H ILE 36 | 4.59789 | 1 | 0.840843976 |
|  | H SER 33 | 4.43323 | 2 | 1.681513906 |
|  | I DG 49 | 4.14653 | 3 | 3.294286728 |
|  | H ALA 35 | 4.4182 | 3 | 2.448784113 |
| ALA 15 |  |  |  |  |
|  | J DA -55 | 4.56346 | 1 | 0.854615986 |
|  | J DT -54 | 3.59597 | 3 | 1.742177248 |
| LYS 4 |  |  |  |  |
|  | J DT 20 | 4.49338 | 4 | 3.020516872 |
|  |  |  |  |  |

**Supplementary Table S3 AII iii.** Hydrogen bonds formed between the highest ranked docking orientation of the unmodified AII (See Figure S5 AII) tip structure and the NCP. Molecule names are as they are defined in the PDB structure 1KX5

| **Tip Residue** | **Interacting Partner**  **<Molecule Name> <Residue Name> <Residue Number>** | **Hydrogen Bond Energy**  **(kJ/mol)** | **Role** | **Donor**  **Atom** | **Acceptor**  **Atom** |
| --- | --- | --- | --- | --- | --- |
| ALA 1 |  |  |  |  |  |
|  | I DT -26 | 25 | don | N | O2P |
| ARG 2 |  |  |  |  |  |
|  | I DC -25 | 9 | don | NE | O2P |
|  | I DC -25 | 12.325 | don | NH2 | O2P |
| LYS 4 |  |  |  |  |  |
|  | J DG 21 | 15.25 | don | NZ | N7 |
|  | J DG 21 | 6.3 | don | NZ | O6 |
| ARG 8 |  |  |  |  |  |
|  | J DG -56 | 16.425 | don | NH1 | O2P |
| LYS 9 |  |  |  |  |  |
|  | B GLY 99 | 18.75 | don | NZ | O |
| LYS 14 |  |  |  |  |  |
|  | H LYS 31 | 19.55 | don | NZ | O |
|  | I DG 49 | 15.825 | don | NZ | O5* |

**Supplementary Table S3 AIII i**. All contacts found between the highest ranked docking orientation of the unmodified AIII tip structure and the NCP (See Figure S5 AIII). Molecule names are as they are defined in the PDB structure 1KX5.

| **Tip Residue** | **Interacting Partner**  **<Molecule Name> <Residue Name> <Residue Number>** | **Distance (Å)** | **Number of Contacts** | **Total Number of Contacts** |
| --- | --- | --- | --- | --- |
| ALA 1 |  |  |  | 142 |
|  | A ALA 88 | 3.50604 | 1 |  |
|  | I DC -25 | 2.05709 | 13 |  |
|  | I DC -24 | 2.15983 | 19 |  |
|  | A SER 86 | 2.42278 | 19 |  |
|  | A SER 87 | 1.92681 | 44 |  |
|  | A GLN 85 | 1.44823 | 46 |  |
| ARG 2 |  |  |  | 37 |
|  | I DT -26 | 3.90853 | 1 |  |
|  | B GLY 102 | 3.14575 | 9 |  |
|  | I DC -25 | 1.74057 | 27 |  |
| SER 10 |  |  |  | 22 |
|  | I DG 49 | 3.88147 | 1 |  |
|  | I DA 51 | 3.9846 | 1 |  |
|  | I DT 50 | 2.74691 | 20 |  |
| LYS 14 |  |  |  | 189 |
|  | I DG 48 | 3.94428 | 1 |  |
|  | H TYR 34 | 3.77118 | 1 |  |
|  | H MET 56 | 3.20762 | 3 |  |
|  | H TYR 39 | 3.26805 | 6 |  |
|  | I DG 49 | 1.83685 | 14 |  |
|  | H SER 33 | 2.57967 | 22 |  |
|  | H ALA 35 | 1.77285 | 64 |  |
|  | H ILE 36 | 1.67094 | 78 |  |
| ALA 15 |  |  |  | 53 |
|  | J DA -55 | 3.6496 | 1 |  |
|  | J DA -53 | 3.40127 | 2 |  |
|  | H TYR 39 | 2.95674 | 18 |  |
|  | J DT -54 | 2.5071 | 32 |  |
| THR 3 |  |  |  | 44 |
|  | I DT -26 | 3.40202 | 2 |  |
|  | I DC -25 | 1.99435 | 42 |  |
| LYS 9 |  |  |  | 29 |
|  | I DT 50 | 3.813 | 2 |  |
|  | I DT 53 | 3.05358 | 5 |  |
|  | I DG 52 | 2.74987 | 7 |  |
|  | I DA 51 | 2.77702 | 15 |  |
|  |  |  |  |  |
| GLY 12 |  |  |  | 5 |
|  | J DA -53 | 3.31529 | 2 |  |
|  | J DT -54 | 2.89881 | 3 |  |
| GLY 13 |  |  |  | 15 |
|  | H ILE 36 | 3.4017 | 3 |  |
|  | I DT 50 | 2.7285 | 12 |  |
|  |  |  |  |  |
| THR 6 |  |  |  | 21 |
|  | I DT -26 | 2.54342 | 8 |  |
|  | I DC -25 | 1.64952 | 13 |  |
| LYS 4 |  |  |  | 19 |
|  | J DA -57 | 2.58539 | 9 |  |
|  | J DG -56 | 2.27548 | 10 |  |
| ARG 8 |  |  |  | 80 |
|  | J DA -57 | 2.09964 | 15 |  |
|  | J DG -56 | 2.04249 | 65 |  |
|  |  |  |  |  |

**Supplementary Table S3 AIII ii.** Hydrophobic contacts found between the highest ranked docking orientation of the unmodified AIII tip structure and the NCP (See Figure S5 AIII). Molecule names are as they are defined in the PDB structure 1KX5.

| **Tip Residue** | **Interacting Partner** | **Distance (Å)** | **Number of Contacts** | **Strength of interaction** |
| --- | --- | --- | --- | --- |
|  | **<Molecule Name> <Residue Name> <Residue Number>** |  |  |  |
|  |  |  |  |  |
| THR 3 |  |  |  |  |
|  | I DT -26 | 4.91295 | 1 | 0.714820027 |
|  | I DC -25 | 3.75126 | 3 | 1.501987934 |
| ARG 8 |  |  |  |  |
|  | J DG -56 | 4.5392 | 1 | 0.82900399 |
| LYS 9 |  |  |  |  |
|  | I DT 53 | 4.65649 | 1 | 0.817403972 |
|  | I DA 51 | 4.13378 | 3 | 2.552258968 |
| GLY 12 |  |  |  |  |
|  | J DT -54 | 4.863 | 1 | 0.734799981 |
| GLY 13 |  |  |  |  |
|  | I DT 50 | 4.06891 | 1 | 0.747903824 |
| LYS 14 |  |  |  |  |
|  | H SER 33 | 4.78907 | 1 | 0.72945416 |
|  | H ALA 35 | 4.04191 | 1 | 0.695980787 |
|  | H ILE 36 | 3.75494 | 5 | 4.329352856 |
| ALA 15 |  |  |  |  |
|  | H TYR 39 | 4.92924 | 1 | 0.701952517 |
|  | J DT -54 | 4.67899 | 2 | 1.472603083 |
|  |  |  |  |  |

**Supplementary Table S3 AIII iii.** Hydrogen bonds formed between the highest ranked docking orientation of the unmodified AIII (See Figure S5 AIII) tip structure and the NCP. Molecule names are as they are defined in the PDB structure 1KX5

| **Tip Residue** | **Interacting Partner** | **Hydrogen Bond Energy**  **(kJ/mol)** | **Role** | **Donor**  **Atom** | **Acceptor**  **Atom** |
| --- | --- | --- | --- | --- | --- |
|  | **<Molecule Name> <Residue Name> <Residue Number>** |  |  |  |  |
| ALA 1 |  |  |  |  |  |
|  | I DC -24 | 12 | don | N | O1P |
| THR 3 |  |  |  |  |  |
|  | I DC -25 | 23.85 | don | N | O2P |
| LYS 4 |  |  |  |  |  |
|  | J DG -56 | 10.4 | don | NZ | O2P |
| THR 6 |  |  |  |  |  |
|  | I DC -25 | 25 | don | OG1 | O2P |
| LYS 14 |  |  |  |  |  |
|  | I DG 49 | 13.5 | don | NZ | O2P |

**Supplementary Table S3 BI i**. All contacts found between the highest ranked docking orientation of the hyper-acetylated BI tip structure and the NCP (See Figure S5 BI). Molecule names are as they are defined in the PDB structure 1KX5.

| **Tip Residue** | **Interacting Partner** | **Distance (Å)** | **Number of Contacts** | **Total Contacts** |
| --- | --- | --- | --- | --- |
|  | **<Molecule Name> <Residue Name> <Residue Number>** |  |  |  |
|  |  |  |  |  |
| ALA 1 |  |  |  | 7 |
|  | J DA -63 | 3.84417 | 1 |  |
|  | J DC -64 | 3.19269 | 6 |  |
| THR 3 |  |  |  | 4 |
|  | J DC -65 | 3.81123 | 1 |  |
|  | J DC -64 | 3.60055 | 3 |  |
| ALA 7 |  |  |  | 31 |
|  | I DA 68 | 3.97688 | 1 |  |
|  | G LYS 124 | 2.98422 | 6 |  |
|  | I DT 69 | 2.39598 | 7 |  |
|  | G LYS 126 | 2.08719 | 17 |  |
| ALY 9 |  |  |  | 34 |
|  | I DT 70 | 3.95031 | 1 |  |
|  | I DA 68 | 3.48801 | 4 |  |
|  | I DT 69 | 1.92809 | 29 |  |
| ALY 14 |  |  |  | 8 |
|  | I DT 67 | 3.91142 | 1 |  |
|  | I DA 68 | 3.02627 | 7 |  |
| ALY 4 |  |  |  | 185 |
|  | I DG 64 | 3.02193 | 4 |  |
|  | I DA 66 | 2.90057 | 7 |  |
|  | I DG 65 | 2.87712 | 8 |  |
|  | J DA -63 | 2.45536 | 15 |  |
|  | I DA 68 | 2.57251 | 21 |  |
|  | I DT 67 | 2.44549 | 28 |  |
|  | J DC -65 | 2.19043 | 30 |  |
|  | J DC -64 | 1.69868 | 72 |  |
| THR 6 |  |  |  | 74 |
|  | I DA 68 | 2.50798 | 8 |  |
|  | G LYS 126 | 2.98996 | 10 |  |
|  | J DT -66 | 2.34515 | 13 |  |
|  | I DT 69 | 2.52854 | 18 |  |
|  | J DC -65 | 1.94365 | 25 |  |
| GLN 5 |  |  |  | 81 |
|  | I DT 69 | 2.35378 | 14 |  |
|  | I DA 68 | 1.61161 | 67 |  |
|  |  |  |  |  |
| ARG 2 |  |  |  | 146 |
|  | G LYS 74 | 2.40667 | 19 |  |
|  | J DA -63 | 1.86493 | 22 |  |
|  | J DC -64 | 0.99872 | 105 |  |
| ARG 8 |  |  |  | 105 |
|  | G SER 125 | 2.31876 | 39 |  |
|  | G LYS 126 | 1.80871 | 66 |  |

**Supplementary Table S3 BI ii**. Hydrophobic contacts found between the highest ranked docking orientation of the hyper-acetylated BI tip structure and the NCP (See Figure S5 BI). Molecule names are as they are defined in the PDB structure 1KX5.

| **Tip Residue** | **Interacting Partner** | **Distance (Å)** | **Number of Contacts** | **Strength of interaction** |
| --- | --- | --- | --- | --- |
|  | **<Molecule Name> <Residue Name> <Residue Number>** |  |  |  |
|  |  |  |  |  |
| ALY 4 |  |  |  |  |
|  | I DA 68 | 3.72098 | 1 | 0.189541668 |
|  | J DA -63 | 3.88692 | 1 | 0.397923082 |
|  | I DT 67 | 3.76931 | 4 | 2.306883812 |
|  | J DC -64 | 3.93769 | 4 | 3.662033319 |
| THR 6 |  |  |  |  |
|  | I DT 69 | 4.85426 | 1 | 0.738295972 |
| ALA 7 |  |  |  |  |
|  | G LYS 124 | 4.48609 | 2 | 1.621968031 |
|  | G LYS 126 | 4.65658 | 2 | 1.567335963 |
| ALY 9 |  |  |  |  |
|  | I DT 69 | 3.50404 | 2 | 0.743317306 |
| ARG 8 |  |  |  |  |
|  | G SER 125 | 4.02443 | 3 | 2.473639488 |
|  | G LYS 126 | 4.01833 | 3 | 3.413038254 |
| ARG 2 |  |  |  |  |
|  | J DC -64 | 3.83676 | 4 | 2.929586887 |
| GLN 5 |  |  |  |  |
|  | I DA 68 | 3.70643 | 4 | 2.422707081 |

**Supplementary Table S3 BI iii**. Hydrogen bonds formed between the highest ranked docking orientation of the hyper-acetylated BI tip structure and the NCP (See Figure S5 BI). Molecule names are as they are defined in the PDB structure 1KX5.

| **Tip Residue** | **Interacting Partner** | **Hydrogen Bond Energy**  **(kJ/mol)** | **Role** | **Donor**  **Atom** | **Acceptor**  **Atom** |
| --- | --- | --- | --- | --- | --- |
|  | **<Molecule Name> <Residue Name> <Residue Number>** |  |  |  |  |
| ARG 2 |  |  |  |  |  |
|  | J DA -63 | 11.15 | don | O2P | NE |
|  | J DA -63 | 6.8 | don | O2P | NH2 |
| ALY 4 |  |  |  |  |  |
|  | J DC -64 | 21.5 | don | NZ | O4* |

**Supplementary Table S3 CI i**. Total contacts found between the highest ranked docking orientation of the active CI tip structure and the NCP (See Figure S5 CI). Molecule names are as they are defined in the PDB structure 1KX5.

| **Tip Residue** | **Interacting Partner**  **<Molecule Name> <Residue Name> <Residue Number>** | **Distance (Å)** | **Number of Contacts** |  |
| --- | --- | --- | --- | --- |
|  |  |  |  | **Total Contacts** |
|  |  |  |  |  |
| ARG 2 |  |  |  | 34 |
|  | I DC -14 | 3.97185 | 1 |  |
|  | I DG 62 | 3.15265 | 1 |  |
|  | A ARG 53 | 3.6953 | 1 |  |
|  | I DG -15 | 2.40353 | 31 |  |
| THR 3 |  |  |  | 17 |
|  | I DG 62 | 3.82605 | 1 |  |
|  | I DG -16 | 3.31558 | 3 |  |
|  | I DG -15 | 2.17683 | 13 |  |
| ALA 7 |  |  |  | 6 |
|  | I DG 61 | 3.62858 | 1 |  |
|  | J DA -57 | 3.13159 | 2 |  |
|  | J DG -56 | 2.97797 | 3 |  |
| ALY 9 |  |  |  | 243 |
|  | J DA -57 | 3.63457 | 1 |  |
|  | H ALA 55 | 3.53645 | 1 |  |
|  | H SER 53 | 3.44284 | 3 |  |
|  | J DA -55 | 2.53595 | 8 |  |
|  | G PRO 80 | 2.46213 | 15 |  |
|  | G ILE 79 | 2.13123 | 26 |  |
|  | H SER 52 | 1.84464 | 31 |  |
|  | J DG -56 | 1.60922 | 48 |  |
|  | H LYS 54 | 1.29734 | 110 |  |
| SER 10 |  |  |  | 32 |
|  | A SER 87 | 3.83236 | 1 |  |
|  | A MET 90 | 2.34574 | 14 |  |
|  | H LYS 54 | 2.50807 | 17 |  |
|  | ALY 14 |  |  | 22 |
|  | H MET 56 | 3.73838 | 1 |  |
|  | J DT -54 | 2.81428 | 8 |  |
|  | H SER 53 | 2.288 | 13 |  |
| ALA 1 |  |  |  | 6 |
|  | I DT 63 | 3.59222 | 2 |  |
|  | I DG 62 | 3.17511 | 4 |  |
|  |  |  |  |  |
|  |  |  |  |  |
|  |  |  |  |  |
|  |  |  |  |  |
| THR 11 |  |  |  | 59 |
|  | A SER 87 | 3.44467 | 3 |  |
|  | B GLY 99 | 2.20324 | 5 |  |
|  | H SER 57 | 3.51301 | 6 |  |
|  | B PHE 100 | 2.85277 | 13 |  |
|  | H LYS 54 | 2.14569 | 32 |  |
| ARG 8 |  |  |  | 17 |
|  | J DA -57 | 2.98299 | 5 |  |
|  | J DG -56 | 2.56481 | 12 |  |
| THR 6 |  |  |  | 35 |
|  | I DA 60 | 2.60432 | 7 |  |
|  | J DA -57 | 2.93227 | 9 |  |
|  | I DG 61 | 2.64339 | 19 |  |
| GLN 5 |  |  |  | 39 |
|  | I DG 61 | 2.64475 | 9 |  |
|  | J DT 20 | 2.1456 | 9 |  |
|  | J DT 19 | 1.93758 | 21 |  |
| GLY 12 |  |  |  | 9 |
|  | H SER 53 | 2.63992 | 9 |  |
| M3L 4 |  |  |  | 109 |
|  | I DG 62 | 1.96944 | 24 |  |
|  | I DG 61 | 2.00371 | 85 |  |

**Supplementary Table S3 CI ii**. Hydrophobic contacts found between the highest ranked docking orientation of the active CI tip structure and the NCP (See Figure S5 CI). Molecule names are as they are defined in the PDB structure 1KX5.

| **Tip Residue** | **Interacting Partner** | **Distance (Å)** | **Number of Contacts** | **Strength of interaction** |
| --- | --- | --- | --- | --- |
|  | **<Molecule Name> <Residue Name> <Residue Number>** |  |  |  |
|  |  |  |  |  |
| ARG 2 |  |  |  |  |
|  | I DG -15 | 4.12914 | 1 | 0.99237448 |
| GLN 5 |  |  |  |  |
|  | J DT 19 | 4.70929 | 1 | 0.761239052 |
| ALY 9 |  |  |  |  |
|  | G PRO 80 | 4.34206 | 1 | 0.943175972 |
|  | G ILE 79 | 3.68255 | 2 | 0.583888471 |
|  | H SER 52 | 3.92123 | 2 | 1.307318211 |
|  | J DG -56 | 4.00494 | 3 | 2.633101463 |
|  | H LYS 54 | 3.83175 | 6 | 10.7713747 |
| THR 11 |  |  |  |  |
|  | H LYS 54 | 4.95472 | 1 | 0.698112011 |
|  | H SER 57 | 4.78531 | 1 | 0.765875995 |
|  | B PHE 100 | 4.46403 | 3 | 2.410233974 |
| GLY 12 |  |  |  |  |
|  | H SER 53 | 4.09741 | 1 | 0.973770857 |
| ALY 14 |  |  |  |  |
|  | J DT -54 | 4.39309 | 1 | 0.922764003 |
|  | H SER 53 | 4.27101 | 1 | 0.971596003 |
| SER 10 |  |  |  |  |
|  | A MET 90 | 4.52306 | 2 | 1.53367424 |
| M3L 4 |  |  |  |  |
|  | I DG 61 | 4.64862 | 5 | 3.613871813 |

**Supplementary Table S3 CI iii**. Hydrogen bonds formed between the highest ranked docking orientation of the active CI tip structure and the NCP (See Figure S5 CI). Molecule names are as they are defined in the PDB structure 1KX5.

| **Tip Residue** | **Interacting Partner** | **Hydrogen Bond Energy**  **(kJ/mol)** | **Role** | **Donor**  **Atom** | **Acceptor**  **Atom** |
| --- | --- | --- | --- | --- | --- |
|  | **<Molecule Name> <Residue Name> <Residue Number>** |  |  |  |  |
| THR 3 |  |  |  |  |  |
|  | I DG -15 | 15.05 | don | N | O1P |
| GLN 5 |  |  |  |  |  |
|  | J DT 20 | 18.45 | don | NE2 | O1P |
| ALY 9 |  |  |  |  |  |
|  | J DG -56 | 11.925 | don | N | O1P |

**Supplementary Table S3 DI i**. Total contacts found between the highest ranked docking orientation of the inactive DI tip structure and the NCP (See Figure S5 DI). Molecule names are as they are defined in the PDB structure 1KX5.

| **Tip Residue** | **Interacting Partner** | **Distance (Å)** | **Number o**  **f Contacts** | **Total Contacts** |
| --- | --- | --- | --- | --- |
|  | **<Molecule Name> <Residue Name> <Residue Number>** |  |  |  |
| LYS 4 |  |  |  | 20 |
|  | I DC 16 | 3.48736 | 1 |  |
|  | I DG 14 | 3.62762 | 2 |  |
|  | J DG -11 | 3.61624 | 3 |  |
|  | I DC 15 | 2.85751 | 14 |  |
| LYS 14 |  |  |  | 72 |
|  | I DA 10 | 3.32662 | 1 |  |
|  | J DT -10 | 3.39448 | 1 |  |
|  | J DC -8 | 3.82237 | 1 |  |
|  | I DC 11 | 3.36774 | 4 |  |
|  | J DT -9 | 1.50935 | 65 |  |
| ARG 2 |  |  |  | 42 |
|  | I DC 16 | 3.54271 | 3 |  |
|  | J DG -11 | 1.98522 | 14 |  |
|  | J DT -12 | 2.51082 | 25 |  |
| THR 3 |  |  |  | 86 |
|  | I DC 16 | 2.66331 | 3 |  |
|  | J DA -13 | 3.19193 | 3 |  |
|  | J DG -11 | 2.52436 | 3 |  |
|  | I DC 15 | 2.05073 | 21 |  |
|  | J DT -12 | 1.34929 | 56 |  |
| ALA 1 |  |  |  | 81 |
|  | E ARG 69 | 3.30723 | 4 |  |
|  | I DT 17 | 1.9137 | 13 |  |
|  | I DC 16 | 1.80184 | 64 |  |
| GLY 13 |  |  |  | 5 |
|  | J DT -9 | 3.29301 | 5 |  |
| ALA 15 |  |  |  | 20 |
|  | J DT -9 | 2.82779 | 8 |  |
|  | J DC -8 | 2.39676 | 12 |  |
| THR 6 |  |  |  | 59 |
|  | J DT -10 | 2.87727 | 9 |  |
|  | J DG -11 | 1.75331 | 50 |  |
| ARG 8 |  |  |  | 62 |
|  | J DT -9 | 2.93226 | 9 |  |
|  | J DT -10 | 1.80998 | 53 |  |
| GLN 5 |  |  |  | 46 |
|  | J DG -11 | 2.23154 | 46 |  |

**Supplementary Table S3 DI ii**. Hydrophobic contacts found between the highest ranked docking orientation of the inactive DI tip structure and the NCP (See Figure S5 DI). Molecule names are as they are defined in the PDB structure 1KX5.

| **Tip Residue** | **Interacting Partner** | **Distance (Å)** | **Number of Contacts** | **Strength of interaction** |
| --- | --- | --- | --- | --- |
|  | **<Molecule Name> <Residue Name> <Residue Number>** |  |  |  |
|  |  |  |  |  |
| ALA 1 |  |  |  |  |
|  | I DC 16 | 4.22384 | 1 | 0.990463972 |
| THR 3 |  |  |  |  |
|  | I DC 15 | 3.86694 | 1 | 0.359499991 |
|  | J DT -12 | 4.89235 | 1 | 0.723060012 |
| GLN 5 |  |  |  |  |
|  | J DG -11 | 4.79812 | 1 | 0.725848615 |
| THR 6 |  |  |  |  |
|  | J DG -11 | 3.68571 | 1 | 0.010980769 |
| GLY 13 |  |  |  |  |
|  | J DT -9 | 4.95694 | 1 | 0.662573695 |
| ARG 2 |  |  |  |  |
|  | J DT -12 | 4.24304 | 2 | 1.65845418 |
| LYS 4 |  |  |  |  |
|  | I DC 15 | 4.44813 | 2 | 1.609645367 |
| ARG 8 |  |  |  |  |
|  | J DT -10 | 4.3919 | 2 | 1.593999982 |
| LYS 14 |  |  |  |  |
|  | J DT -9 | 4.44383 | 3 | 2.464577675 |

**Supplementary Table S3 DI iii**. Hydrogen bonds formed between the highest ranked docking orientation of the inactive DI tip structure and the NCP (See Figure S5 DI). Molecule names are as they are defined in the PDB structure 1KX5.

| **Tip Residue** | **Interacting Partner** | **Hydrogen Bond Energy**  **(kJ/mol)** | **Role** | **Donor**  **Atom** | **Acceptor**  **Atom** |
| --- | --- | --- | --- | --- | --- |
|  | **<Molecule Name> <Residue Name> <Residue Number>** |  |  |  |  |
| ALA 1 |  |  |  |  |  |
|  | I DT 17 | 13.3 | don | N | O1P |
| ARG 2 |  |  |  |  |  |
|  | J DG -11 | 6.7 | don | NE | O2P |
|  | J DG -11 | 16.025 | don | NH2 | O2P |
| THR 6 |  |  |  |  |  |
|  | J DG -11 | 21.85 | don | OG1 | O3* |
| ARG 8 |  |  |  |  |  |
|  | J DT -10 | 11.35 | don | NH1 | O3* |


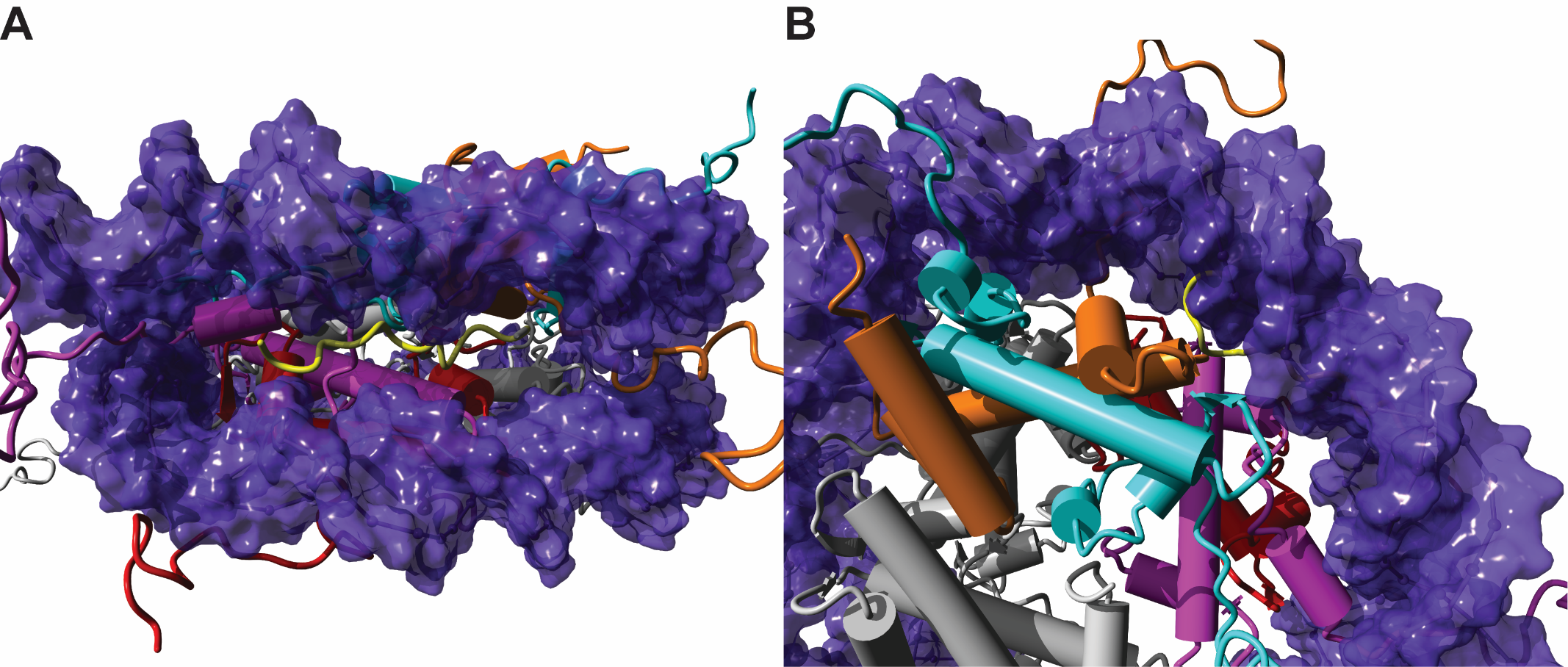


**Supplementary Figure S9. A.** Side view of the binding of the active tip structure with the NCP. **B.** Top view of the binding of the active tip structure with the NCP. Histone H3 is indicated in magenta, H4 is indicated in red, H2A is cyan, H2B is indicated in orange, other octamer components are in indicated in gray and the DNA is indicated in violet. The tail tip is indicated in yellow, with its c-terminal end indicated in gray.

Molecular graphics created with YASARA (www.yasara.org) and POVRay (www.povray.org)


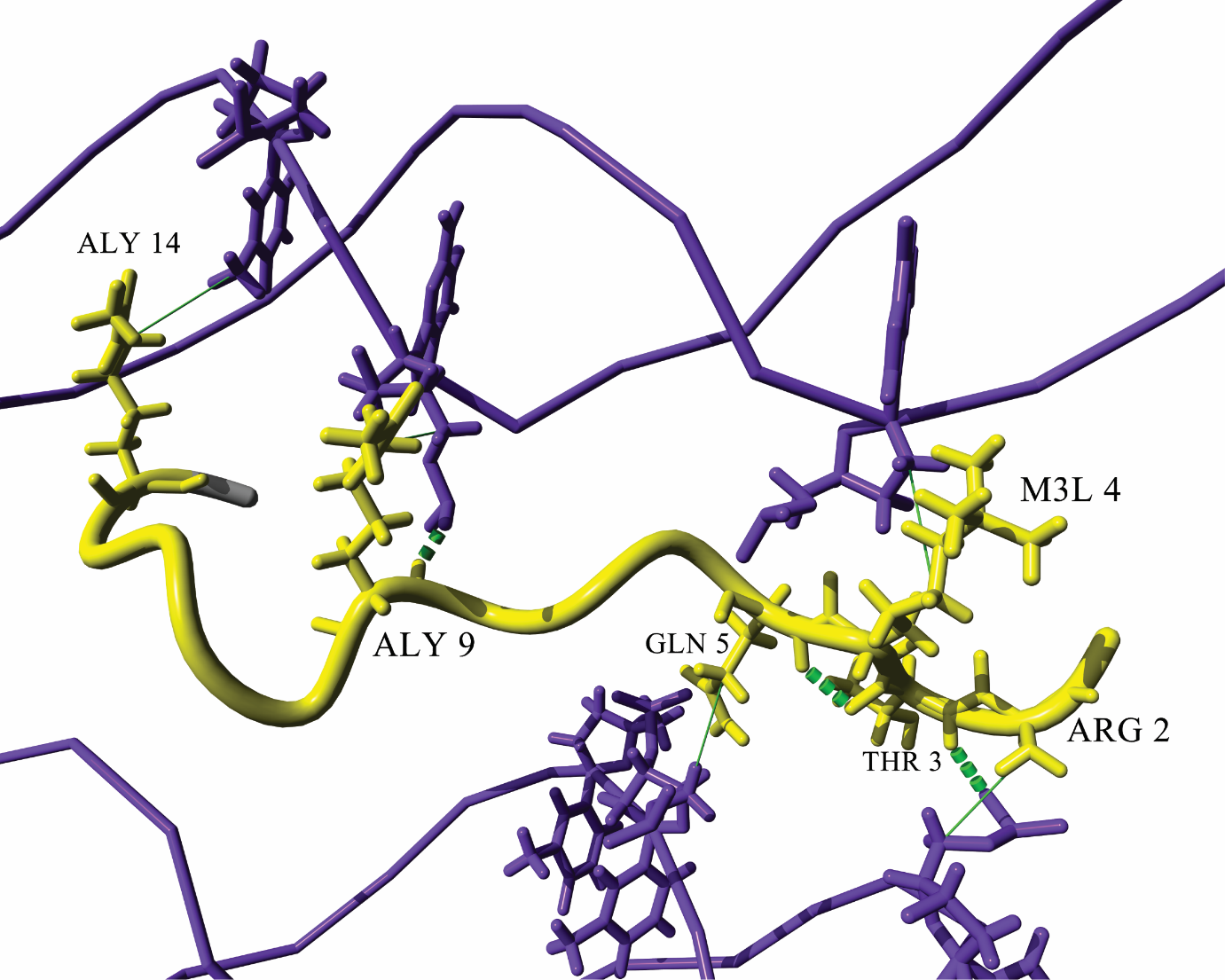


**Supplementary Figure S10.** Interaction active tip structure (yellow) with the DNA. Only Thr3 and Aly9 forms hydrogen bonds with the nucleotide backbones of the the DNA, with hydrophobic interactions dominate the interactions of the tip with the DNA. The DNA is indicated in violet. Hydrophobic interactions are represented as solid green lines and hydrogen bonds are represented by dashed green lines.

Molecular graphics created with YASARA (www.yasara.org) and POVRay (www.povray.org)


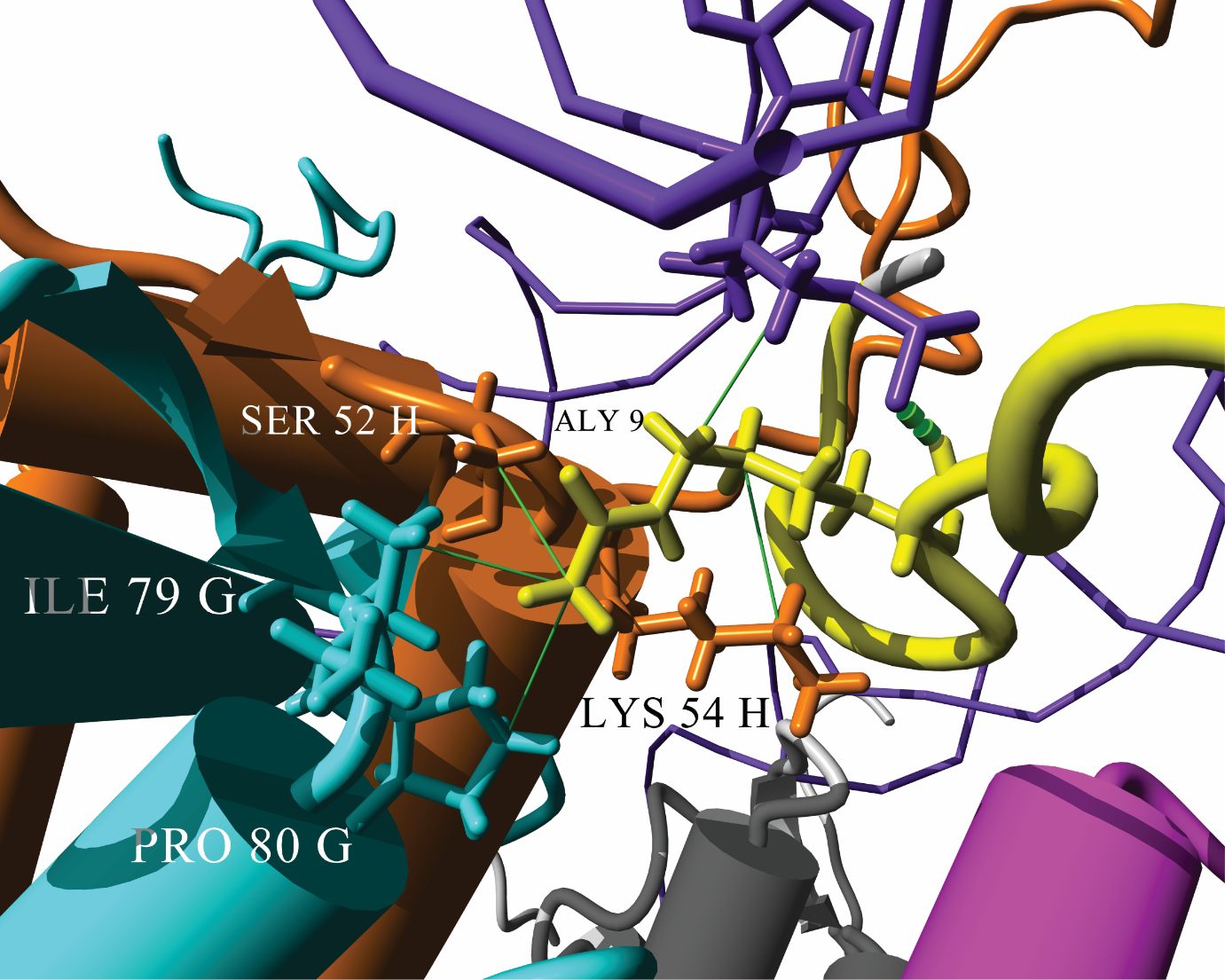


**Supplementary Figure S11.** Binding of the side chain of the acetylated Lys9 into the hydrophobic pocket formed by Ser52 and Lys54 from histone H2B, Ile79 and Pro80 from histone H2A and a nucleotide from the DNA. Histone H3 is indicated in magenta, H4 is indicated in red, H2A is cyan, H2B is indicated in orange, other octamer components are in indicated in gray and the DNA is indicated in violet. The tail tip is indicated in yellow, with its c-terminal end indicated in gray.

Molecular graphics created with YASARA (www.yasara.org) and POVRay (www.povray.org)


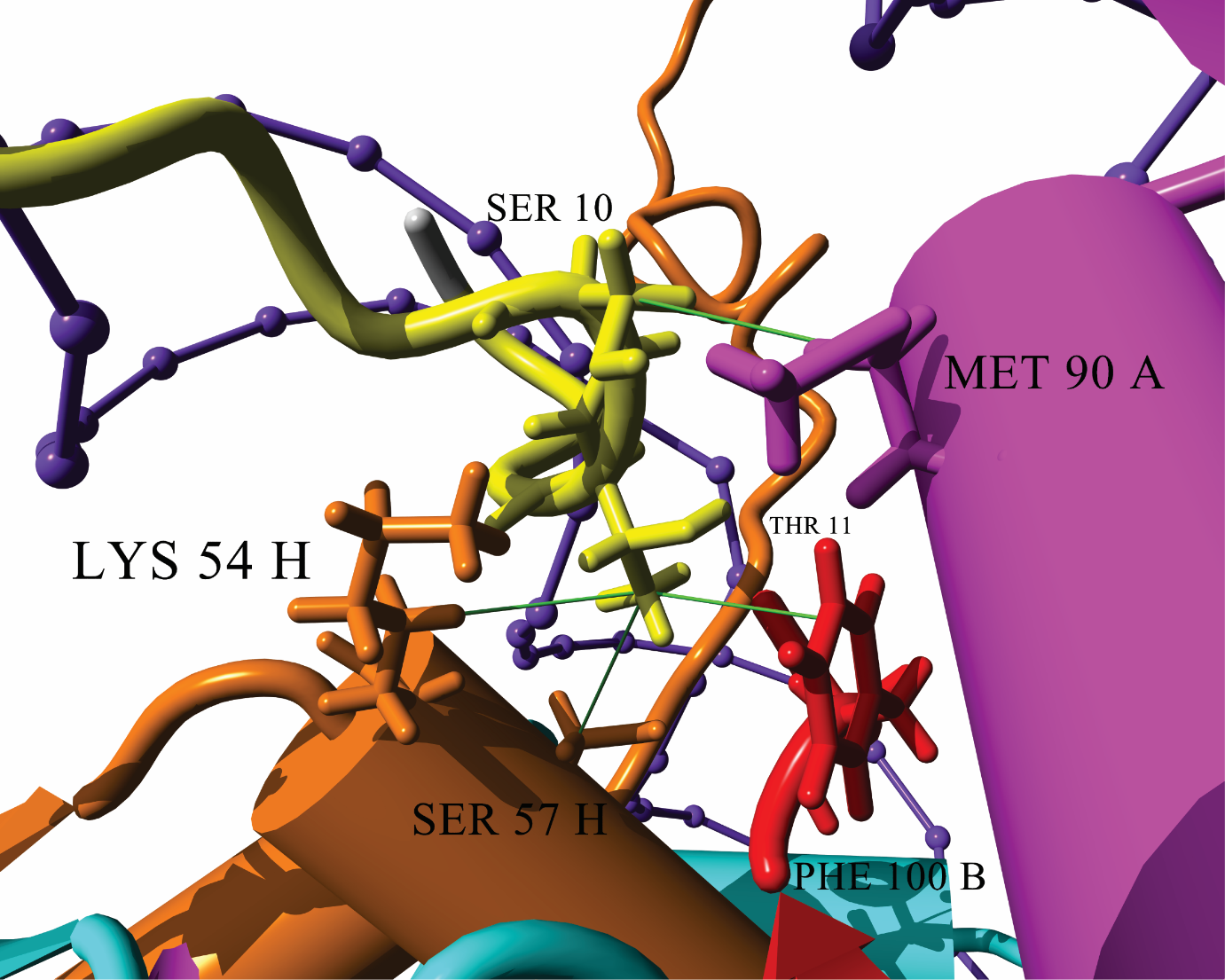


**Supplementary Figure S12.** The binding of Thr11 into the hydrophobic pocket formed Lys54 and Ser57 from histone H2B and Phe100 from histone H4. The adjacent Ser10 also makes a hydrophobic interaction with Met90 of histone H3. Histone H3 is indicated in magenta, H4 is indicated in red, H2A is cyan, H2B is indicated in orange, other octamer components are in indicated in gray and the DNA is indicated in violet. Hydrophobic interactions are represented by solid green lines.

Molecular graphics created with YASARA (www.yasara.org) and POVRay (www.povray.org)


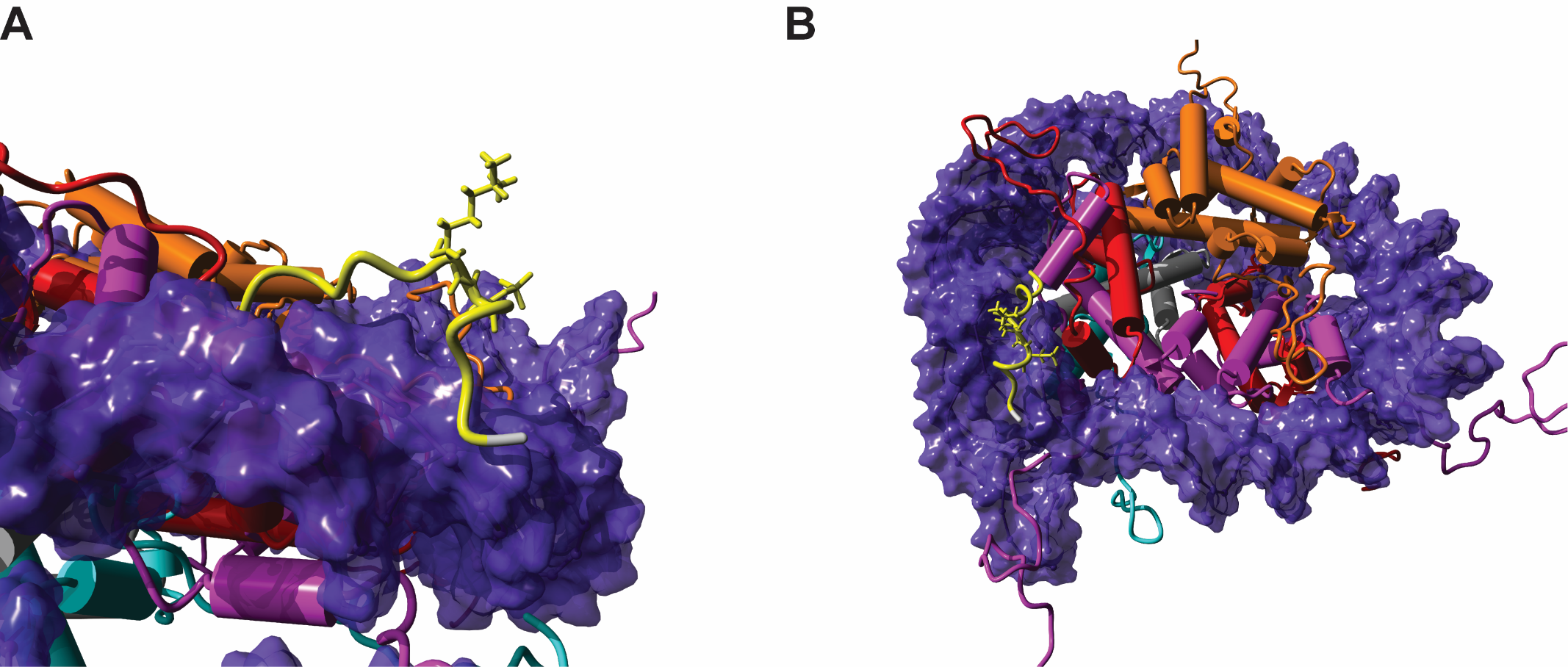


**Supplementary Figure S13. A.** The inactive tip structure binds exclusively over the top DNA gyre with its n – terminal slotted into the minor groove with the di – methylated Lys9 and phosphorylated Ser10 pointing away from the NCP. **B.** The inactive tip binds over the exit point of the other H3 molecule (molecule E), as opposed to in the vicinity of the exit point of molecule A, as found with the other tip structures. The DNA is indicated in violet. Hydrophobic interactions are represented as solid green lines and hydrogen bonds are represented by dashed green lines.

Molecular graphics created with YASARA (www.yasara.org) and POVRay ([www.povray.org](http://www.povray.org))


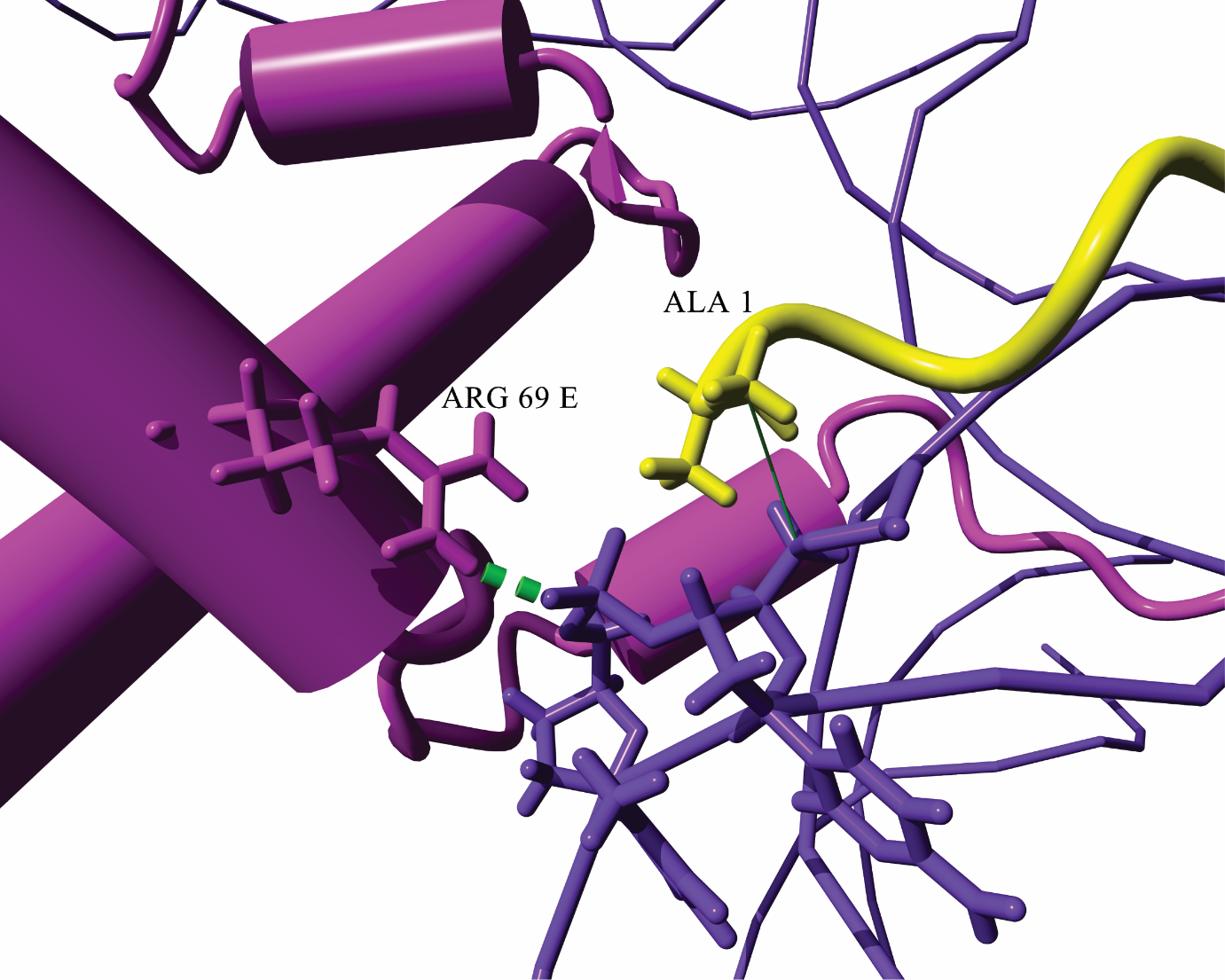


**Supplementary Figure S14.** The side chain of the acetylated Lys14 side chain makes a hydrophobic contact with Ser53 of histone H2B and a nucleotide. H2A is indicated in cyan, H2B is indicated in orange, other octamer components are in indicated in gray and the DNA is indicated in violet. Hydrophobic interactions are represented by solid green lines.

Molecular graphics created with YASARA (www.yasara.org) and POVRay (www.povray.org)


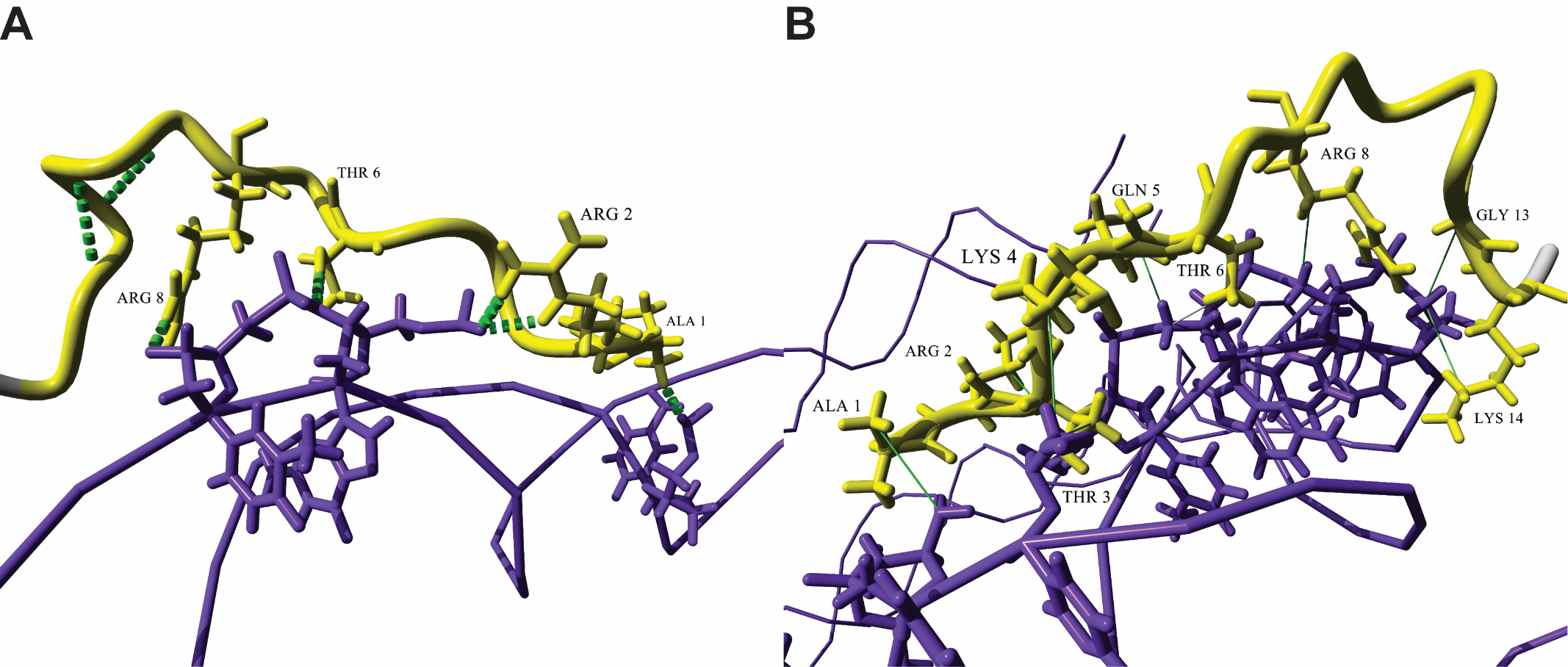


**Supplementary Figure S15. A.** Ala1, Arg2, Thr6 and Arg8 of the inactive tip structure forms hydrogen bonds with the backbone of the nucleotides in the binding site. **B.** Ala1, Arg2, Thr3, Lys4, Gln5, Thr6, Arg8, Gly13 and Lys14 also make hydrophobic contacts with the nucleotides in the binding site. The DNA is indicated in violet. Hydrophobic interactions are represented as solid green lines and hydrogen bonds are represented by dashed green lines.

Molecular graphics created with YASARA (www.yasara.org) and POVRay ([www.povray.org](http://www.povray.org))

**Supplementary Figure S16.** Super-imposition of the docked tail tips (green) for the (A) unmodified tail, (B) the hyper-acetylated tail, (C) the active tail and (D) the inactive tail and the structure of the tips after 10 ns MD simulations (yellow).


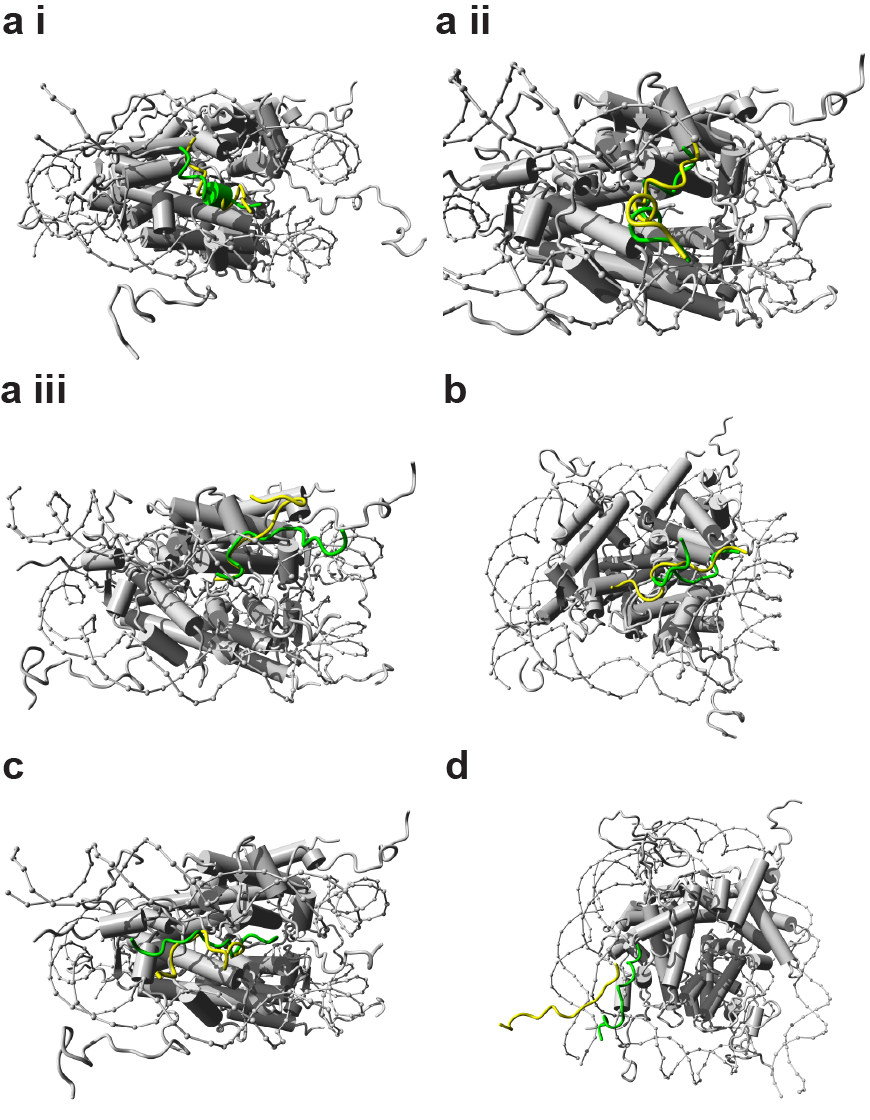


**Supplementary Figure S17.** α – Carbon RMSD of the (A) unmodified tail tips, (B) the hyper-acetylated tail tips, (C) the active tail tips and (D) the inactive tail tips during 10 ns MD simulations of the top docking poses obtained


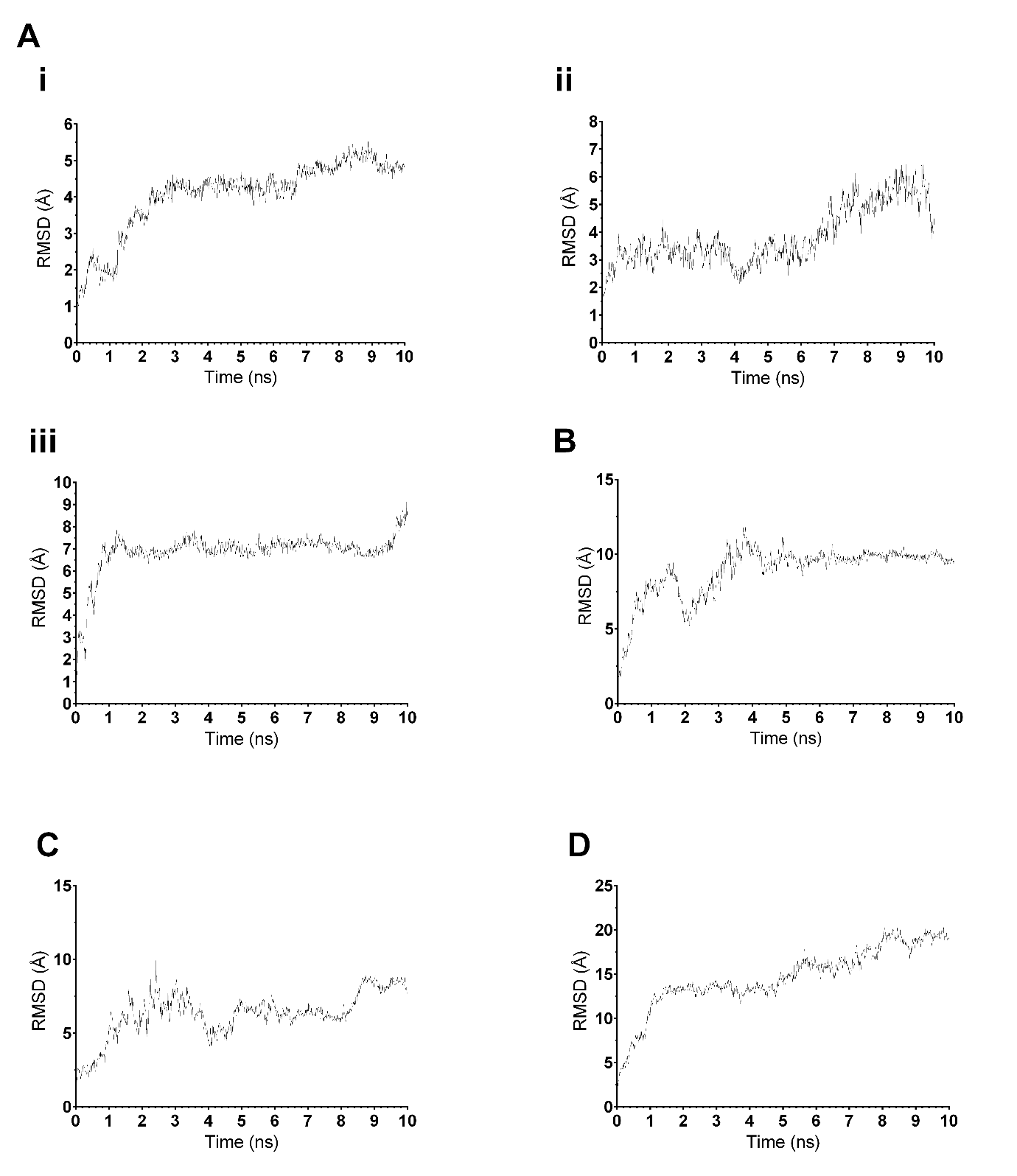


**Supplementary Figure S18.** Reach of the active (A and B) and inactive (C and D) tails with a tetra-nucleosomal model relative to the acidic patch on each nucleosome, based MD reach data of the tails (See Figure 8). The acidic residues of the acidic patch is indicated in red.


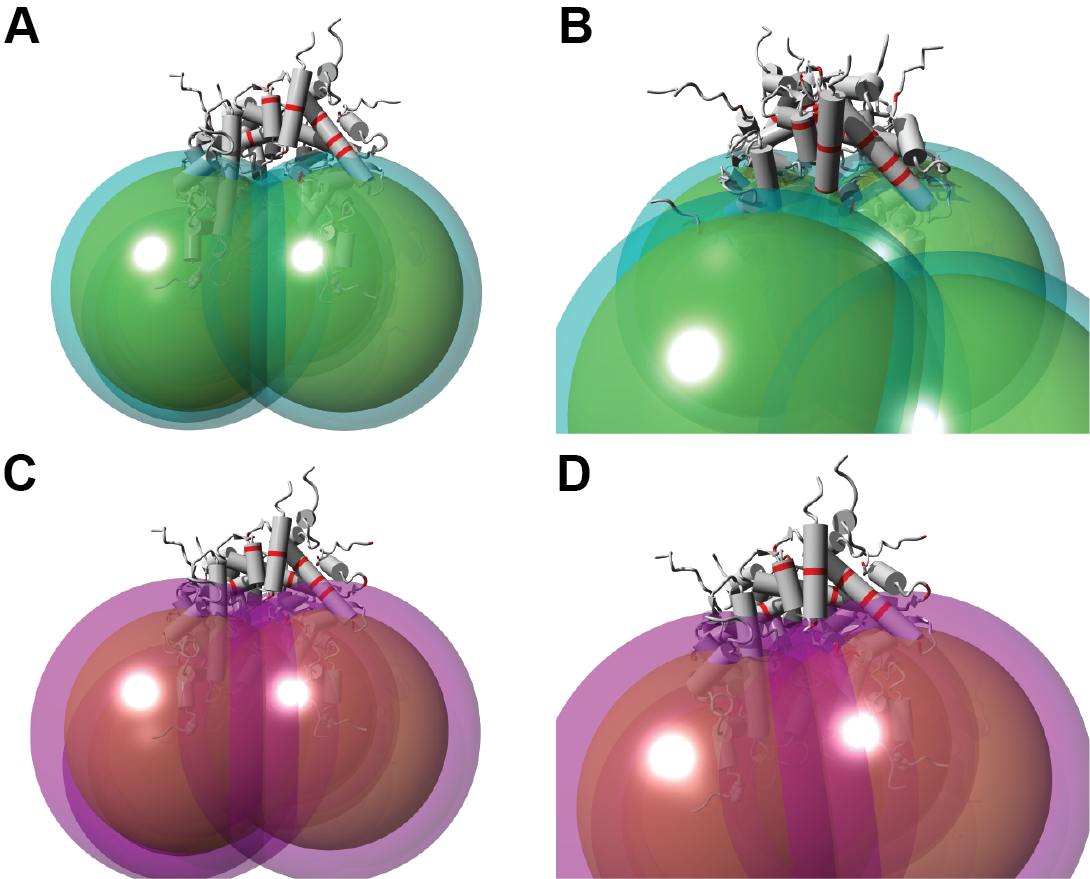


Supplementary References

1. Chou,P.Y. and Fasman,G.D. (1974) Prediction of protein conformation. *Biochemistry*, **13**, 222-245.

2. Cole,C., Barber,J.D. and Barton,G.J. (2008) The Jpred 3 secondary structure prediction server. *Nucleic Acids Research*, **36**, W197-W201.

3. Meiler,J., Müller,M., Zeidler,A. and Schmäñschke,F. (2001) Generation and evaluation of dimension-reduced amino acid parameter representations by artificial neural networks. *Journal of Molecular Modeling*, **7**, 360-369.

4. Petersen,B., Petersen,T., Andersen,P., Nielsen,M. and Lundegaard,C. (2009) A generic method for assignment of reliability scores applied to solvent accessibility predictions. *BMC Structural Biology*, **9**, 51.

5. Mooney,C. and Pollastri,G. (2009) Beyond the Twilight Zone: Automated prediction of structural properties of proteins by recursive neural networks and remote homology information. *Proteins*, **77**, 181-190.

6. Pollastri,G., Martin,A., Mooney,C. and Vullo,A. (2007) Accurate prediction of protein secondary structure and solvent accessibility by consensus combiners of sequence and structure information. *BMC Bioinformatics*, **8**, 201.

7. Pollastri,G. and McLysaght,A. (2004) Porter: a new, accurate server for protein secondary structure prediction. *Bioinformatics*, **21**, 1719-1720.

8. Rost,B. (1996) [31] PHD: Predicting one-dimensional protein structure by profile-based neural networks. In Russell,F.D. (ed.), *Methods in Enzymology*

*Computer Methods for Macromolecular Sequence Analysis*. Academic Press, pp. 525-539.

9. Rost,B., Yachdav,G. and Liu,J. (2004) The PredictProtein server. *Nucleic Acids Research*, **32**, W321-W326.

10. Ouali,M. and King,R.D. (2000) Cascaded multiple classifiers for secondary structure prediction. *Protein Science*, **9**, 1162-1176.

11. Cheng,J., Randall,A.Z., Sweredoski,M.J. and Baldi,P. (2005) SCRATCH: a protein structure and structural feature prediction server. *Nucleic Acids Research*, **33**, W72-W76.

12. Pollastri,G., Przybylski,D., Rost,B. and Baldi,P. (2002) Improving the prediction of protein secondary structure in three and eight classes using recurrent neural networks and profiles. *Proteins*, **47**, 228-235.

13. Combet,C., Blanchet,C., Geourjon,C. and Deléage,G. (2000) NPS@: Network Protein Sequence Analysis. *Trends in Biochemical Sciences*, **25**, 147-150.

14. McGuffin,L.J., Bryson,K. and Jones,D.T. (2000) The PSIPRED protein structure prediction server. *Bioinformatics.*, **16**, 404-405.

15. Wu,H., Min,J., Lunin,V.V., Antoshenko,T., Dombrovski,L., Zeng,H., Allali-Hassani,A., Campagna-Slater,V.r., Vedadi,M., Arrowsmith,C.H. *et al.* (2010) Structural Biology of Human H3K9 Methyltransferases. *PLoS ONE*, **5**, e8570.

16. Yap,K.L., Li,S., Muñoz-Cabello,A.M., Raguz,S., Zeng,L., Mujtaba,S., Gil,J., Walsh,M.J. and Zhou,M.M. Molecular Interplay of the Noncoding RNA ANRIL and Methylated Histone H3 Lysine 27 by Polycomb CBX7 in Transcriptional Silencing of INK4a. Molecular cell 38(5), 662-674. 6-11-2010.

17. Kaustov,L., Ouyang,H., Amaya,M., Lemak,A., Nady,N., Duan,S., Wasney,G.A., Li,Z., Vedadi,M., Schapira,M. *et al.* (2011) Recognition and Specificity Determinants of the Human Cbx Chromodomains. *Journal of Biological Chemistry*, **286**, 521-529.

18. Zeng,L., Zhang,Q., Li,S., Plotnikov,A.N., Walsh,M.J. and Zhou,M.M. (2010) Mechanism and regulation of acetylated histone binding by the tandem PHD finger of DPF3b. *Nature*, **466**, 258-262.

19. Macdonald,N., Welburn,J.P.I., Noble,M.E.M., Nguyen,A., Yaffe,M.B., Clynes,D., Moggs,J.G., Orphanides,G., Thomson,S., Edmunds,J.W. *et al.* Molecular Basis for the Recognition of Phosphorylated and Phosphoacetylated Histone H3 by 14-3-3. Molecular cell 20(2), 199-211. 10-28-2005.
